# Multilayer networks of plasmid genetic similarity reveal potential pathways of gene transmission

**DOI:** 10.1038/s41396-023-01373-5

**Published:** 2023-02-09

**Authors:** Julie Teresa Shapiro, Alvah Zorea, Aya Brown Kav, Vicente J. Ontiveros, Itzhak Mizrahi, Shai Pilosof

**Affiliations:** 1 Department of Life Sciences, Ben-Gurion University of the Negev, Be’er Sheva, Israel; 2 National Institute of Biotechnology in the Negev, Ben-Gurion University of the Negev, Be’er Sheva, Israel; 3 Program for Mathematical Genomics, Department of Systems Biology, Columbia University Irving Medical Center, New York, NY USA; 4 Institute of Aquatic Ecology, University of Girona, Girona, Spain; 5 The Goldman Sonnenfeldt School of Sustainability and Climate Change, Ben-Gurion University of the Negev, Be’er Sheva, Israel; 6 Present Address: Epidemiology and Surveillance Support Unit, University of Lyon, French Agency for Food, Environmental and Occupational Health and Safety (ANSES), Lyon, France

**Keywords:** Microbial ecology, Theoretical ecology, Microbiology

## Abstract

Antimicrobial resistance (AMR) is a significant threat to public health. Plasmids are principal vectors of AMR genes, significantly contributing to their spread and mobility across hosts. Nevertheless, little is known about the dynamics of plasmid genetic exchange across animal hosts. Here, we use theory and methodology from network and disease ecology to investigate the potential of gene transmission between plasmids using a data set of 21 plasmidomes from a single dairy cow population. We constructed a multilayer network based on pairwise plasmid genetic similarity. Genetic similarity is a signature of past genetic exchange that can aid in identifying potential routes and mechanisms of gene transmission within and between cows. Links between cows dominated the transmission network, and plasmids containing mobility genes were more connected. Modularity analysis revealed a network cluster where all plasmids contained a *mobM* gene, and one where all plasmids contained a beta-lactamase gene. Cows that contain both clusters also share transmission pathways with many other cows, making them candidates for super-spreading. In support, we found signatures of gene super-spreading in which a few plasmids and cows are responsible for most gene exchange. An agent-based transmission model showed that a new gene invading the cow population will likely reach all cows. Finally, we showed that edge weights contain a non-random signature for the mechanisms of gene transmission, allowing us to differentiate between dispersal and genetic exchange. These results provide insights into how genes, including those providing AMR, spread across animal hosts.

## Introduction

Antimicrobial resistance (AMR) is a significant threat to human and animal health globally [1]. Livestock may serve as a reservoir of antibiotic-resistant bacteria due to the widespread use of antibiotics in the agricultural sector for prophylaxis and growth promotion [2, 3]. This trend is likely to continue due to increasing demand for animal products and the intensification of livestock production globally [4]. AMR from livestock can spread into the environment, including soils and water bodies [5–8] and contaminate food products, reaching humans [9].

The spread of antibiotic resistance genes within and between species happens primarily via plasmids [10]. Plasmids are mobile genetic elements, often circular and ranging from thousands to hundreds of thousands of base pairs long, that can propagate independently of their host’s chromosome. While they can be found in archaea and eukaryotes, they are most well-known in bacteria [11]. Plasmids allow bacteria to adapt to their environment by carrying accessory genes, such as those for antibiotic [12–14], or heavy metal resistance [14, 15], which are beneficial under particular environmental conditions. Given the relevance of plasmids to the spread of AMR [16, 17], it is essential to understand how they disperse in their natural habitats between their animal hosts and interact with each other. While plasmids are one of the principal vehicles for horizontal gene transfer (HGT) between bacteria, genetic exchange also occurs between plasmids, for example via homologous recombination [11, 18]. This results in gene transfer between plasmids, which we term plasmid-HGT (pHGT). Due to pHGT, many plasmids appear to be mosaic. Mosaic plasmids can incorporate genetic material from multiple plasmids hosted by distantly related bacterial species, although this varies significantly by host taxonomy [19–21].

Livestock can be reservoirs of plasmids containing AMR genes [22–25]. More generally, ruminants, such as cattle, host diverse microbial communities, particularly in the rumen. Rumen microbes allow cattle to digest otherwise indigestible plant matter [26, 27]. While research on plasmids traditionally focused on those that are clinically relevant [28], advances in metagenomics and sequencing technology have enabled the exploration of the plasmidome, all plasmids within a given sampled environment, including the bovine rumen [29, 30]. This approach has revealed that the bovine rumen plasmidome is diverse, and differs more between individuals compared to the bacterial microbiome [29, 30]. Based on the similarity of their open-reading frames (ORFs) to those in bacteria, the plasmids of the bovine rumen appeared most commonly associated with Firmicutes and Bacteroidetes [29, 30]. Many are mosaic, including some showing evidence of cross-phyla genetic exchange [30]. Rumen plasmids are enriched for functions related to digestion and metabolism, as well as plasmid functions such as mobilization and replication, but the majority of ORFs have unknown functions [29, 30]. Similar findings were also reported in other ecosystems, such as the rat cecum, where cryptic plasmids have also been found to dominate the plasmidome of [31].

Genetic exchange and dispersal may leave a signature of genetic similarity within a population, which can be detected using network analysis [32–36]. Thus far, networks of plasmid genetic similarity have primarily been used to analyze broad population structures among plasmid types and classify them into taxonomic units [33, 34, 37]. Network analyses have revealed gene sharing across geography, habitat type, and to some extent host phylogeny [34, 38, 39]. Several studies identified plasmids that provide a bridge between otherwise unconnected bacterial communities [18, 40]. Hence, plasmid genetic similarity networks may allow us to use signatures of past events within a plasmid population to help identify potential pathways for future transmission. This application, which is critical in the context of AMR, remains unexplored.

Because plasmids are infectious agents, disease ecology theory and methodology can prove beneficial to understanding the relationship between network structure and transmission dynamics [41–44]. Using plasmid similarity networks to understand mechanisms of gene spread is tantamount to using parasite-sharing networks as a proxy for potential parasite transmission across multi-species host communities in disease ecology [42, 45], or networks that describe contacts between individuals. Essentially, any “transmission network” describes potential pathways for pathogen transmission, and there are multiple ways to estimate these pathways [44]. In the case of gene transmission across plasmids, this approach requires that plasmid genetic information is collected within the same system. However, nearly all studies used plasmid sequences originating from diverse and geographically distant environments because they were mined from databases [33, 34, 37, 39, 40]. Using plasmids from different systems is inadequate for determining pathways of genetic exchange or dispersal on finer spatial and temporal scales; for instance, between and within animal hosts. One study used plasmid similarity networks constructed from F-type plasmids from livestock farms (cows, pigs, or sheep) and water [46]. The plasmid networks and communities were structured by their broad ecological niche (farm vs. water), demonstrating the potential limits of plasmid dispersal and genetic exchange across environments [46]. Although the data were collected in a natural system, samples from animals were pooled by site, impeding the estimation of plasmid spread between individual hosts.

Here, we leverage a data set of the rumen plasmidome of dairy cows in a single population. We aim to identify signatures of genetic exchange between plasmids and potential pathways for gene transmission within and between cows. To address this goal, we use an ecological multilayer network framework, which explicitly harnesses variation in network structure across multiple data layers [47]. We capture signatures of genetic similarity within and between individual cows (layers). By adopting analytical approaches and terminology from disease ecology, we interpret the data in light of gene transmission. Specifically, we look for signatures of super-spreading at the level of both plasmids and cows, whereby a few plasmids (or cows) are responsible for the majority of transmission [48].

We find that plasmid genetic similarity networks are dominated by links between cow hosts. The transmission network is highly fragmented into clusters of plasmids (i.e., modules) with a highly heterogeneous size distribution, pointing to the dominant role of between-host transmission in shaping the genetic signature of this plasmid population. Such heterogeneity also indicates potential super-spreading at the level of both plasmids and cows. We further found that plasmids with the same AMR genes, though rare in our data set, formed independent network clusters (modules).

Modeling showed that network structure determined the extent of gene transmission. By investigating signatures of genetic similarity in the network, we can understand how plasmids interact within and between animal hosts, providing insights into the mechanisms by which AMR genes can spread.

## Results

### The multilayer network is dominated by an interlayer, cow-to-cow connectivity

**Fig. 1:**
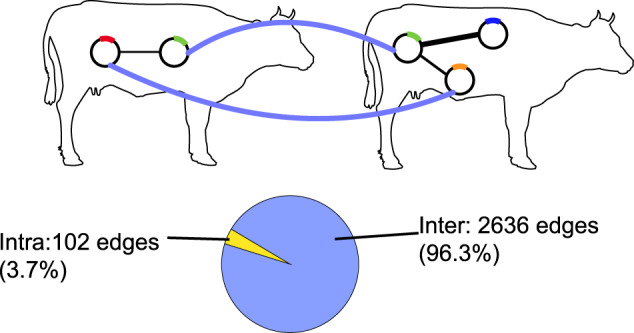
A plasmidome multilayer network definition. Cows are layers and physical nodes are plasmids. The same plasmid can occur in different cows (e.g., the green plasmid). Intralayer (black) and interlayer (blue) links are weighted and defined based on sequence similarity (see Methods). The pie chart shows that the network is dominated by interlayer edges.

We constructed a weighted, multilayer network between plasmids. In our multilayer network, each cow is a layer, and the nodes within each layer are plasmids (Fig. 1). Intralayer links were calculated as the genetic similarity between plasmids within a layer based on sequence alignments (Methods). A prominent feature of multilayer networks is interlayer links, which connect nodes between layers and encode ecological processes that operate between the layers [47]. We defined the interlayer links using the same measure as intralayer links (Fig. 1), allowing us to simultaneously detect signatures of gene exchange within and between cows. Using the same definition is also advantageous because it places intra- and inter-layer processes on the same scale, avoiding a-priori biases of network metrics towards processes operating on either type of edge [36, 47, 49].

Our network included 1344 plasmids in 21 cows. Of these plasmids, 92% were detected in only a single cow. Because plasmids can occur in more than one cow, the total number of nodes in the network exceeds the number of plasmids. Following standard terminology [47, 50], we define plasmids as “physical nodes” and plasmid-layer combinations as “state nodes”, indicating that the same plasmid can be in a different ecological state; for instance, contributing differently to gene exchange in different cows. Our network included 1514 state nodes. The maximum number of cows in which an individual plasmid was detected was 13. The number of plasmids per cow ranged from 1 to 175, with a mean of 72. While pairs of plasmids (i.e., network links) could have multiple alignments between them, 2728 of the 2738 links contained only a single alignment. Overall, the network was very sparse, with only 0.2% of the potential links realized.

We asked whether links were primarily within or between cows to gauge the importance of plasmid interactions between hosts. Most edges in the network were inter-layer (Fig. 1). However, a comparison of raw edge counts is biased by the vastly different number of possible intra- and inter-layer edges (87,002 vs. 1,058,339, respectively). Therefore, we also compared the proportion of realized intra- and interlayer links out of all possible ones (i.e., density). The density of inter-layer edges was twice as high as intra-layer edges (0.25% vs. 0.12%). Therefore, there is a much stronger signature of genetic exchange between cows compared to within them.

We tested the effect of cow identity on connectivity by comparing the observed network to 1000 randomized networks in which we shuffled the identity of cows (hereafter, “shuffled networks”). The observed network had a significantly higher density of intra- (*p* < 0.001) and inter-layer edges (*p* < 0.001), and a higher density (*p* < 0.001) than the shuffled networks (Supplementary Fig. 1). This indicates that despite the sparse nature of the network, it is still more connected and with a larger contribution of inter-layer edges compared to intra-layer than expected at random. Overall, the network was dominated by interlayer connectivity, suggesting that gene exchange is more likely between plasmids from different cows than within a cow.

### Skewed plasmid contribution to gene exchange implies super-spreading

We can use the intra- and interlayer connectivity to test if specific plasmids contribute disproportionately to gene transmission and exchange by examining the distribution of their degree (number of plasmids to which a plasmid is connected) and the distribution of links to cows. Both distributions were highly skewed (skewness = 10.5 and 10.4, respectively) (Supplementary Fig. 2), with most plasmids having a few links and a few plasmids having many links to both other plasmids and cows. A skewed distribution indicates that few plasmids may be responsible for most of the transmission and interactions in the network. This pattern is in line with super-spreading theory from disease ecology that consistently finds that a few hosts (here plasmids) are responsible for the majority of parasite (here an AMR gene) transmission [48, 51] (Supplementary Information).

### The network is characterized by asymmetric and nonrandom pathways of gene transmission

Although the plasmid similarity network is highly sparse, it may still contain major pathways of gene exchange. In epidemiology and disease ecology, areas of potential transmission in networks can be detected using community detection algorithms, loosely referred to as modularity [41, 52]. Modularity is a mesoscale property in which parts of the network are denser compared to others [53]. We detected modules using Infomap, an algorithm based on the movement of a random walker on the network. Infomap is explicitly designed for multilayer networks and also measures the amount of flow contained within each node (the total flow across state nodes in the network sums to 1) [54–56]. Flow measurement is particularly suitable for our purposes because it is directly related to the idea of gene exchange [56]. Modules, therefore, represent high-level potential pathways of transmission.

**Fig. 2:**
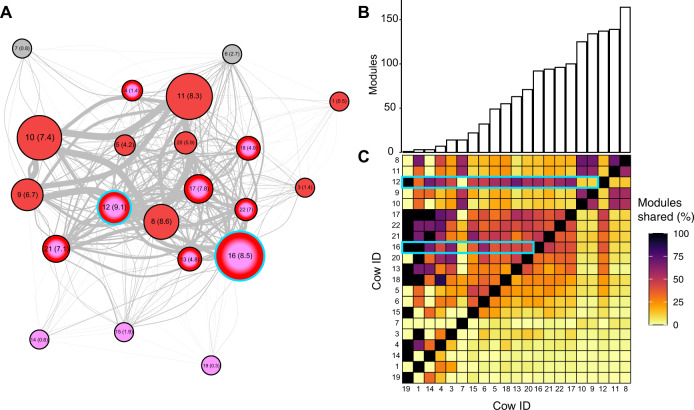
Pathways of gene transmission are related to network structure. **A** A layer-perspective representation of the entire network. Nodes are cows, their size is proportional to the number of intralayer edges within each cow. Node labels indicate the cow ID and in parentheses the relative strength calculated as number of interlayer edges a cow has divided by the total number of interlayer edges in the network. An edge indicates that at least one interlayer edge connects two cows, and their width is proportional to the total number of interlayer edges between cows. Nodes in red, pink, or both indicate cows that appear in the largest module, the beta-lactamase module or in both, respectively. **B** Bar plot of the number of modules per cow. The number of modules per cow ranged from 1 to 164, with a median of 63. **C** The percent of modules shared between cows. Each cell is calculated as the number of modules that cow *j* (columns) shares with *i* (rows), divided by the total number of modules that *j* has. This measure results in an asymmetric matrix because the proportion is calculated with respect to one of the cows in the pair and cows have different numbers of modules. Bars in **B** correspond to the matrix columns in **C** and are ordered by the number of modules per cow, from lowest (left) to highest (right). The cyan rectangles are examples of two cows that share a high proportion of modules. These two cows are also part of the largest and the beta-lactamase modules (cyan border in **A**). Therefore, they may be super-spreaders of AMR genes.

While the network was highly fragmented with 414 modules, it was significantly less so than the shuffled networks, which had, on average 83% more modules (723–797, mean = 760, *p* < 0.001) (Supplementary Fig. 3A). As with the node-level pattern, this mesoscale topology was highly skewed: while most modules in the observed network were small, with an average of 3.3 plasmids across 3.4 cows, one exceptionally large module encompassed 12 plasmids and 16 cows (*≈*4 times the average number of plasmids and cows in a module). This module included 215 links, accounting for 7.9% of all the links in the network, most of which were inter-layer (*n* = 205) and intra-modular (*n* = 206). Hence, it represents a potential major pathway of transmission and genetic exchange between plasmids, particularly across different hosts (Fig. 2A). Nodes within this largest module encompassed 8% of the flow, which is an order of magnitude more than the mean flow per module (0.2%) (Z score = 13.3, *p* < 10^*−*5^). Comparison to the largest module in each shuffled network showed that while the number of unique plasmids (physical nodes) within a module did not differ (*p* = 0.35), the flow in the largest module of the observed network was significantly greater than its counterpart largest modules in the shuffled networks (*p* = 0.003) (Supplementary Fig. 3B). Therefore, more genetic exchange occurs within this large module than expected if plasmids were randomly distributed among cows. Taken together, these results point to a portion of the network where a disproportionately large amount of gene exchange occurred.

The number of modules in which cows were present was skewed (Fig. 2B) but indicated that cows share transmission pathways. To further investigate potential transmission between cows, we calculated module sharing $s_{ij} = \left| {m_i \cap m_j} \right|/m_j$. That is, the number of modules shared between pairs of cows (*m*_*i*_, *m*_*j*_), out of the total modules in cow *m*_*j*_. Module sharing was highly asymmetric, ranging from 0 to 100% (median = 14%) (Fig. 2C). As expected, cows with few modules shared all or most of them. There was a negative correlation between the number of modules a cow hosted and the mean proportion of modules shared with other cows (Kendall’s tau = −0.785, *p* = 7.2e^*−*7^). Thus, a few cows may serve as hubs for many interacting groups of plasmids, linking peripheral cows to transmission pathways. Nevertheless, some cows with many modules still shared a high proportion of them. For instance, four of the five cows with the highest number of modules (125–164 modules) shared 53–69% of their modules with other cows. Thus, there are potential hot spots for plasmid transmission.

The proportion of shared modules between cows was higher than the percentage of shared plasmids, which ranged from 0 to 66.6% with a median of only 0.71% (Supplementary Fig. 4). Nearly half (47.1%) of cow pairs shared no plasmids. While this pattern is partially an artifact of the much larger number of plasmids compared to modules, it also demonstrates that while the cows predominantly do not host identical plasmids, they still share transmission pathways. This observation was further supported by the low correlation between the proportion of shared modules and the proportion of shared plasmids (Kendall’s tau = 0.41, *p* < 2.2e^*−*16^).

To validate the notion of cows as transmission hubs, we compared observed module sharing to that obtained in the shuffled networks. For each cow pair, we calculated a z-score of module sharing (Methods). Out of the 420 possible pairs, 98 shared significantly more (z-score > 1.96), and 194 shared significantly fewer (z-score < *−*1.96) modules than the random expectation. The skew towards fewer pairs of cows sharing many modules is again consistent with potential super-spreading in the network, this time at the cow level. Together, these results illustrate that the signatures of potential super-spreading are observed not only at the plasmid level but also at the cow level.

### Simulations of plasmid-borne gene transmission between cows

We then asked how network structure might affect the spread of a hypothetical AMR plasmid-borne gene through the cow population. To address this, we constructed a stochastic agent-based model of gene transmission. In this model, the gene is initially present in a single plasmid (state node). We randomly chose 20 starting plasmids: 10 highly-connected plasmids that are a part of the largest module and 10 peripheral plasmids from the smallest modules (those containing two state nodes). In each time step, a certain number of plasmid pairs come in contact. If one of the plasmids in the pair has the gene, the probability that the gene is transmitted to the second plasmid is equal to the genetic similarity (edge-weight) between them since research has shown that pHGT is positively correlated with genetic similarity [57]. Bacteria are implicit in our model, and we assume that plasmid contact occurs within bacterial cells. The gene may also be lost from a plasmid with a rate depending on the level of positive selection pressure (higher selection pressure leads to lower loss rates). To determine the trade-offs between plasmid contact and selection pressure, we ran the model with low (10 plasmids in contact), intermediate (100 plasmids in contact), and high (1000 plasmids in contact) total contact rates and accounted for selection pressure through gene loss rates: high (0 loss per capita), intermediate (0.01 per capita), and low (0.1 per capita). We ran the model for 1000 time steps and measured the number of cows to which the AMR gene arrived.

**Fig. 3:**
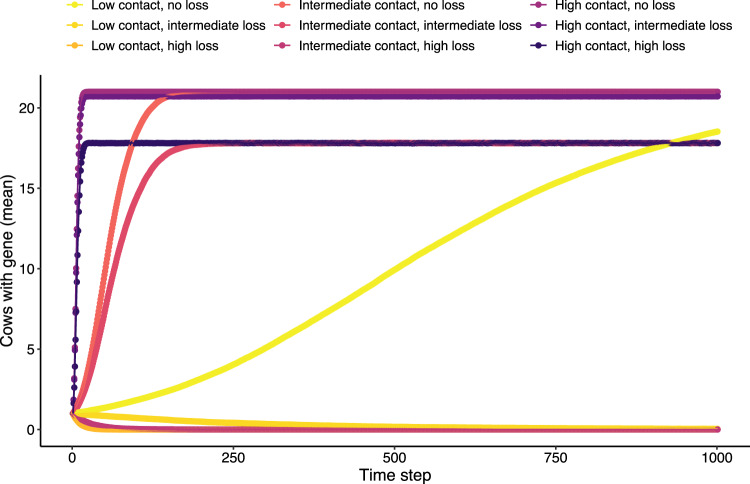
Simulated gene transmission dynamics in a cow population. Results of simulations of gene transmission among cows when the gene originates in a highly-connected plasmid. Each point is the number of cows with the gene at each time step averaged over 300 simulations per plasmid. Contact refers to the contact rate between plasmids. When plasmids encounter each other, and consequently exchange genes, at high rates, the gene is quickly transmitted to all the cow population. See Supplementary Fig. 5 for results of simulations starting with peripheral plasmids.

At high contact rates, the gene quickly dispersed to all cows in approximately the same amount of time at any level of selection pressure (Fig. 3). At intermediate contact, the gene could reach all cows at high or intermediate selection pressure. However, it took, on average, approximately nine times as many time steps compared to the high selection pressure simulations. At low contact, it was only possible for the gene to reach all cows at high selection pressure, but this only occurred in a small percent of simulations (14–15%) and when it did, it required ~900 time steps on average (Fig. 3, Supplementary Table 1).

Patterns of gene transmission were nearly identical when starting in either highly connected or peripheral plasmids (Fig. 3, Supplementary Fig. 5, Supplementary Table 1). Similar dynamics between highly connected or peripheral plasmids may stem from the fact that once a plasmid reaches a well-connected cow, it spreads extremely quickly to others (Fig. 2). For instance, even the five cows that are not a part of the largest module still contained other large modules (9–18 state nodes). Thus, even under low or intermediate selection pressure, between-cow module connectivity may allow genes to rapidly reach all hosts in this population via pHGT if contact between plasmids is high enough. Another possible factor is the small cow population size (*n* = 21) studied here.

### Link weights are indicative of mechanisms underlying plasmid similarity

**Fig. 4:**
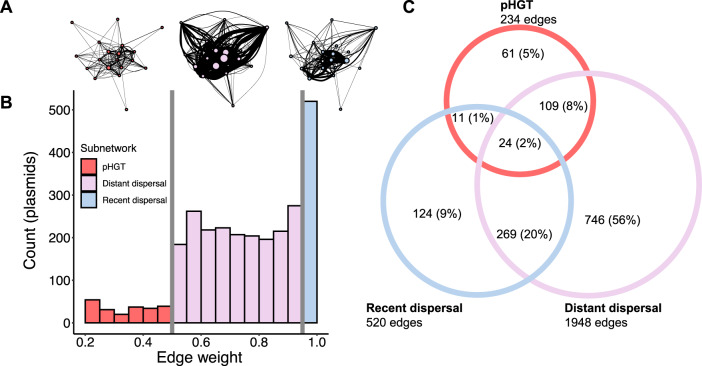
Edge-weight distribution indicating mechanisms of gene exchange. **A** A simplified visualization of each multilayer subnetwork (similar to Fig. 2A). Nodes are cows and their size is proportional to the number of intralayer edges within them. Edges indicate inter-layer edges between cows and their width is proportional to the total number of interlayer edges. **B** The distribution of edge weights in the network has two abrupt breaks (vertical gray lines) at 0.5 and 0.95. **C** Venn diagram depicting the number of plasmids shared between the three subnetworks. Percentages are calculated with respect to the total number of plasmids in the data set. Circle size is illustrative of the number of plasmids and edges in the subnetwork.

So far, we have shown that there are nonrandom signatures of disproportionate contributions of plasmids and cows to gene exchange. However, these patterns do not suggest particular mechanisms by which gene exchange can occur. We hypothesize that such information is contained in the distribution of link weights. Specifically, plasmid dispersal should be manifested by high similarity between plasmids (very strong links), as the two nodes are essentially the same plasmid. In contrast, low link weights will indicate pHGT because a small section of the plasmids’ DNA is shared (e.g., via recombination). The distribution of edge weights in the network displayed two abrupt breaks at 0.5 and 0.95 (Fig. 4A, B). We hypothesize that these breaks correspond to pHGT (edge-weights < 0.5) and dispersal (edge-weights > 0.95). Between these two scenarios lies a third one, which we call “distant dispersal”. In distant dispersal, a plasmid disperses and then undergoes genetic change via mutation or rearrangements by transposons. While it is impossible to pinpoint the particular mechanism of genetic change by pairwise genetic similarity in this scenario, such a mechanism could explain why distant dispersal lies between HGT and dispersal.

The division into subnetworks represents a hypothesis that these mechanisms (pHGT and dispersal) drive the observed edge-weight distribution. The first step to address this hypothesis is to test whether this distribution is non-randomly determined by sequence similarity, which is the pattern resulting from gene exchange. We resampled the alignment length between plasmid pairs without replacement 1000 times, leaving all other values the same. We then recalculated the edge weights on the resampled data set. The two abrupt breaks did not exist in the resampled networks. In addition, the observed edge weight distribution was significantly different from that obtained by resampling (Kolmogorov-Smirnov test, *D* = 0.25*, p* < 0.0001), indicating that the observed distribution is likely the result of biological processes (Supplementary Fig. 6).

We then investigated each gene exchange mechanism by dividing the network into three multilayer subnetworks according to the link weights (Fig. 4A, B). The subnetworks varied in size, with the distant dispersal subnetwork dominating in numbers of plasmids and edges (Fig. 4C). While edges could only belong to one subnetwork, plasmids could belong to multiple subnetworks (Fig. 4C). The pHGT subnetwork had the highest density of intra- (0.50%) and inter-layer edges (0.78%) compared to both the recent dispersal (intra-layer = 0.38%; inter-layer = 0.22%) and distant dispersal (intra-layer = 0.11%; inter-layer = 0.25%) subnetworks. These differences in density point to the importance of pHGT in driving patterns of genetic similarity seen in the network.

### Genes of mobility and antibiotic resistance affect node importance and network structure

We then asked whether plasmid functional traits influence plasmid importance and the modular structure. To do so, we examined the role of plasmid mobility genes, which provide the ability to transfer to new bacterial hosts (Methods). Plasmids that are conjugative (or self-transmissible) code for all the necessary proteins to transfer themselves while those that are mobilizable must use the machinery, particularly the mating pair formation complex, of another element in the cell for transfer [58–60]. Generally, at least half of plasmids are non-mobilizable (neither conjugative nor mobilizable) [58]. We measured the effect of *mob* genes on plasmids’ degree, module flow, and the characteristics of the modules they are found in using Mann-Whitney-Wilcoxon tests. In the data set, 235 plasmids (17.4%) had *mob* genes. We found that plasmids with *mob* genes had a significantly higher degree and greater flow but were not found in a greater number of cows, compared to plasmids without the *mob* genes. Modules containing plasmids with *mob* genes (83 out of 414) were larger, contained more unique plasmids, encompassed more cows, and had a greater flow (Table 1).

**Table 1:** Comparison of node- and module-level metrics between plasmids and modules with (*n* = 235) and without (*n* = 1109) mob genes based on the Mann-Whitney-Wilcoxon test. We show the median values for plasmids or modules with and without *mob* genes, *U*, and *p* value. Significant *p* values (<0.05) are indicated in bold. The magnitude of the differences in these measures is small, possibly due to the low number of plasmids for which mob-related ORFs were detected.

Scale	Measure	Median		*U*	*p* value
		mob present	mob absent		
Plasmid	Degree	3	2	118335	**0.02**
	Flow	0.00042	0.00048	118570	**0.03**
	Cows	1	1	126275	0.11
Module	Unique plasmids	3	3	11728	**0.03**
	Plasmid-cow combination	3	3	11209	**0.006**
	Cows	3	3	11350	**0.01**
	Flow	0.0011	0.00087	11485	**0.02**

All 12 plasmids in the largest module (Fig. 2A) contained a *mobM* gene, known to be widely distributed across plasmids isolated from enterococci, streptococci, and staphylococci. The former two are prevalent within the rumen environment, and consist of known representatives such as *Streptococcus bovis* and *Enterococcus faecalis* [61], which could explain the plasmid presence within this module. A plasmid with a mobility gene is likely to have high dispersal rates between bacterial hosts of these lineages and hence between cows. Therefore, this module could be composed of plasmids recombining with their bacterial hosts’ genomes and among themselves as they occur in the same hosts. It is important to note that plasmid-plasmid interactions could be mediated through the bacterial genome. Therefore, plasmids are not necessarily required to temporally coincide within the same cell to recombine. An alternative hypothesis is that these plasmids belong to a common plasmid ancestor whose multiple copies underwent genetic changes while mobilizing across hosts. Supporting this hypothesis is the fact that 95% of the edges in the module were dispersal edges. Nevertheless, these two scenarios of plasmid recombination and plasmid ancestor modification are not mutually exclusive.

We identified ORFs related to antimicrobial resistance in 12 plasmids (0.9%). Eight were for beta-lactam, three for tetracycline resistance, and one for penicillin-binding protein. Interestingly, plasmids with beta-lactam and tetracycline resistance were separated into two distinct modules that contained no other plasmids. The module containing the beta-lactam-resistant plasmids spanned 11 different cows, making it the third largest in the network in terms of layers (Fig. 2A). Of the edges linking plasmids with beta-lactam resistance, 77% belonged to the distant dispersal subnetwork while the rest belonged to the recent dispersal subnetwork. All the links between the three plasmids with tetracycline resistance corresponded to the distant dispersal network. Thus, plasmid dispersal is likely the primary mechanism for the spread of plasmid-mediated antibiotic resistance between hosts. This suggests that selective pressure for antibiotic resistance genes could promote the dispersal and persistence of these plasmids across cows.

## Discussion

A significant gap in understanding the spread of AMR involves understanding how plasmid-borne AMR genes spread within and between microbial communities and animal hosts. We addressed this gap by drawing upon theory and methods from microbial and disease ecology, using multilayer networks of genetic similarity between plasmids in a population of dairy cows. While plasmid genetic similarity networks have previously been used to classify plasmids [33, 34, 37] and identify the transmission of plasmid-borne genes across environments and geography at evolutionary scales [18, 38, 39], these scales are less relevant for transmission within animal populations. Here we used the signature left by the genetic exchange between plasmids in their patterns of sequence similarity to understand plasmid transmission between hosts at a scale relevant to the transmission of plasmid-mediated antimicrobial resistance.

Specifically, we found evidence for super-spreading, including the highly skewed distributions for node degree at the plasmid level and module sharing at the level of cows. Moreover, we showed that the module that contains plasmids with a *mobM* gene and the module of plasmids with a beta-lactam resistance gene coincide in eight of the 21 cows. These cows could be super-spreaders of beta-lactam resistance because its spillover from the beta-lactam module to the largest module could cause a rapid transmission of the gene across the cow population. Super-spreading has been found in the spread of antimicrobial resistance in both humans [62–64], and cows [65]. Social networks from cows have also shown that most individuals have relatively few links to others, while a few individuals are highly connected, further indicating the potential for super-spreading in the case of an outbreak [66].

Identifying patterns of super-spreading is important because they can guide management and control strategies to prevent the spread of pathogens or antimicrobial resistance and increase the effectiveness of interventions by targeting the most highly-connected individuals or groups in the network [51, 65, 67]. The transmission model we presented is a novel approach in this direction because it links the structure of plasmid similarity networks with gene transmission. While plasmid similarity networks are typically used to discern processes that generated observed structures, our model can be further used to test multiple hypotheses regarding the effect of network structure on potential gene transmission.

The presence of *mob* genes affected plasmid connectivity and network structure. Previous studies also found that conjugative and mobilizable plasmids are more connected than non-mobilizable ones [39, 68]. Plasmids with *mob* genes may have a higher node degree because they can disperse more quickly between bacteria and encounter other plasmids more frequently, especially since mobilizable or conjugative plasmids are more likely to be found in different bacterial families. This was particularly evident in the plasmids of the largest module. In contrast, non-mobilizable plasmids are more likely to be restricted to a single species [34]. However, it is also important to consider that many small plasmids can still spread rapidly in bacterial populations, although currently the mechanisms are not well-understood [68].

Analyzing modularity in our network allowed us to identify potential transmission pathways between hosts. Hosts with similar plasmids are more likely to share AMR genes, as has been shown for pathogen types [42]. The fact that all plasmids with beta-lactam and tetracycline resistance were found in distinct modules supports the idea that modules represent potential gene transmission pathways. This conclusion is strengthened by our observation that plasmid mobility strongly affected the modular structure. The presence of several large modules encompassing a large proportion of cows’ rumen ecosystems in this population suggests that both plasmids and the genes they carry can quickly disperse through this host population. The dynamical model supported this result. Because the connectivity of modules between cows is high, even if a gene appears in a peripheral cow, it will quickly disperse to others.

The network was dominated by inter-layer edges, linking plasmids in different cows. We hypothesize that interlayer connectivity is mediated mainly by between-cow bacterial transfer, while intralayer connectivity is mediated by plasmid conjugation within the rumen of each individual cow. Research on plasmid-mediated antimicrobial resistance in healthcare showed that plasmids often transfer between individuals via a single bacterial strain. At the same time, they tend to transfer between species within the gut microbiome within individuals [62]. Thus, our results could indicate that these plasmids are transferred between cows carried by particularly efficient microbial colonizers. However, plasmids have also been shown to disperse between human hosts independently of the transmission of bacteria [69]. Our plasmidome data do not include the bacterial hosts involved (see below). Therefore, while we cannot test this hypothesis, future studies in this direction may shed light on the relative importance of multilevel HGT and pHGT processes (bacteria movement or plasmid movement).

We used the similarity networks to discern the roles of HGT and dispersal, both of which play an important role in the spread of antimicrobial resistance [62, 69]. Our initial exploration of these new hypotheses showed that the distinctly segmented edge weight distribution was non-random and, thus, was likely driven by biological processes. Nevertheless, the division among pHGT, recent dispersal, and distant dispersal network remains a hypothesis. Further research, via both modeling and experiments, is needed to test this hypothesis by observing rates of pHGT and mutation in plasmid populations and determining under what conditions the edge-weight distribution can be reproduced.

Our results clearly show extensive dispersal of plasmids between cows, but we do not have information on the dispersal mechanism. In healthcare settings, plasmids transferred between patients via healthcare workers or environmental reservoirs [62, 69–71]. In cows, plasmids could be transferred via the saliva during grooming, especially if rumen plasmids were regurgitated during rumination or otherwise found in the mouth. Alternatively, transmission may occur via the fecal-oral route as cows may rub, sniff, or lick the genital area of other individuals [66]. Transmission could also be possible via bioaerosols from feces [72]. Combining our plasmid similarity networks with social networks, using measures such as proximity, allogrooming, or shared space use between cows [43, 66, 73] could provide further insights into how plasmids are transmitted between individuals.

Our study has several limitations. First, we have not explicitly considered the plasmids’ bacterial hosts. Second, we do not have any measures of cow social contact. Third, our data represent only a single snapshot in time; longitudinal time-series analysis could provide further information on the dynamics of plasmid interactions and dispersal [74]. Fourth, our method of calculating plasmid similarity relies on alignments, which might not consider rearrangements in the genomes of plasmids [33]. Finally, we only consider circular plasmids, although linear plasmids can also carry AMR genes (also see Methods) [75]. Despite these limitations, our study provides the first insight into potential gene exchange via plasmid at a relevant spatio-temporal resolution.

In conclusion, genetic similarity networks provide a powerful tool for understanding the transmission potential of plasmids and their genes within host populations. We demonstrated that plasmids are transmitted extensively between individuals within a population of dairy cows, with signatures of super-spreading at the level of both plasmids and cows. Plasmid functions, particularly AMR and mobility, influence the network structure. The genetic similarity between plasmids in this population of cows shows signatures of both dispersal and genetic exchange, providing insights into how plasmid-mediated AMR can spread across hosts.

## Methods

### Study system and initial data processing

We used an existing data set of plasmids sequenced from the rumens of 22 individual Israeli Holstein dairy cows housed on the same farm [30]. This experimental setup includes a typical and standard husbandry diet applied in multiple farms worldwide for intensive farming regimes. This allowed us to have consistent conditions with no known confounding factors. The cows in the study were not treated with antibiotics, as those harm the rumen microbiome on which they depend. Hence, the general expectation of identifying AMR genes was not high a-priori.

Sampling and bio-informatic protocols were previously described [30]. In brief, samples of rumen fluid were obtained from each cow, DNA was extracted, amplified using phi29 polymerase, and sequenced using the paired-end protocol (GAIIX sequencer and HiSeq [Illumina]) [30]. Reads from each cow were first assembled into plasmid contigs using SPAdes [76]. We focused on plasmids as mobile elements while eliminating chromosomal DNA that could skew our analyses. Hence, we used the recycler tool [77] to select only circular plasmids from the contigs to identify closed circular sequences that are not bacterial chromosomes. Nevertheless, even with this stringent approach, the method still identified plasmids ranging 2000–7297 bp, and carrying AMR genes.

The complete plasmid data set contained 8741 plasmid sequences. Each sequence was assigned a name based on the cow in which it was detected. We compared pairwise plasmid sequences using the BLASTn algorithm [30]. We detected identical plasmid sequences sequenced from different cows in the data set by comparing the plasmid length, alignment length, and percent identity. Identical plasmids were those with the same length and 100% identity over 100% of their length with no gaps in the alignment. We then confirmed that these plasmids were identical by aligning them in the software Geneious (v.11). A total of 314 sequences were identified as identical to at least one other sequence in the data set and were consequently grouped in 138 identical plasmids. Each identical plasmid was assembled from 2 to 5 cows, leaving 8565 unique plasmids. We did not perform any further clustering. Each unique plasmid was assigned a node id number (1–8565). Identical plasmids were assigned the same node id. Each cow was also assigned a unique layer id (1–22). Cow number 2 contained no plasmids that matched our criteria and was excluded.

Because contigs were assembled from samples individually, we mapped the reads from each individual cow back to the full set of plasmid contigs using bbmap with the parameter “ambig” set to “all” to determine whether additional plasmids from the data set not detected in the original assembly were present in individual cows. Based on read mapping, we measured the coverage of each plasmid sequence in each cow. We considered plasmids to be present in a cow if they had 100% coverage in that cow.

### Plasmid annotations

We used previously published annotations for plasmid ORFs [30]. These ORFs were annotated by comparing them to the NCBI-NR protein database using a maximum *E* value cut-off 10^*−*5^. For each ORF, the hit with the lowest *E* value was chosen unless it was a hypothetical protein, in which case we chose the next lowest *E* value. If all five of the lowest *E* value hits were hypothetical proteins, the ORF was annotated as hypothetical. Annotated functions were then manually curated into functional categories such as “plasmid”, “phage” and “sugar metabolism” based on their description in the database.

We conducted additional bioinformatic analyses on all plasmids in the data set using the Resistance Gene Identifier (https://github.com/arpcard/rgi). We did not detect additional AMR genes, but those already in our data set were reverified, corroborating our previous analyses [30]. Moreover, for all plasmids in the data set, we predicted the ORFs using Prokka and used KofamScan to identify KEGG orthologies, which resulted in 15 KOs across 40 plasmids. We specifically blasted the plasmids in module #1 (the largest module). Because blast results of entire plasmids had no hits, we also manually looked for conserved domains of the plasmids in module #1 and module #2 (which contained the plasmids with the beta-lactam-carrying plasmids) using the Conserved Domain tool with NCBI (https://www.ncbi.nlm.nih.gov/Structure/cdd/wrpsb.cgi?RID=3FEF4TF8013&mode=all). These results can be found in the Supplementary Information.

### Network construction

We used undirected networks because the directionality of exchange or divergence between plasmids cannot be obtained from sequence similarity alone [37, 78]. For each plasmid pair, we calculated an edge weight as:

$$w_{ij} = \mathop {\sum }\limits_1^k min\left( {\frac{{l_s}}{{l_j}},\frac{{l_s}}{{l_i}}} \right)p_i,$$

where *i* and *j* are aligned plasmids, *l*_*s*_ is the length of the alignment, *l*_*i*_ and *l*_*j*_ are the total lengths of plasmids *i* and *j*, *k* is the number of alignments between the plasmids, and *p*_*i*_ is the percent identity between the plasmids for a given alignment. While plasmid length in the data set ranged from 2000 to 7297 bp (mean = 2751 bp), aligned plasmids generally had a similar length, with 72% between 2000 and 3000 bp.)

Before constructing our networks, we performed a sensitivity analysis to determine the cut-off for plasmid length to include in our analysis. We compared the number of plasmids and alignments (total, intralayer, and interlayer) retained in the data at thresholds for plasmid length (500–3000 bp in increments of 500 bp) and alignment length (20% of the shorter plasmid in a given pair and 20% of the threshold). While the number of intralayer edges was relatively stable at all thresholds, we found that the number of inter-layer edges retained plateaued at a length threshold of 2000 bp while there was virtually no effect of the two alignment thresholds. Based on these results, we restricted our analyses to plasmid sequences > =2000 bp and alignments that covered > = 20% of the length of the shortest plasmid in a pair (Supplementary Fig. 7). Minimum percent identity for alignments was >=70% [30].

### Basic network metrics

We calculated each plasmid’s intra-, inter- and total degree as the number of intra-layer links, inter-layer links, and both, respectively. We tested for skewness in the distribution of degree centrality and layer links using the function skewedness in the package moments [79]. We calculated network density as the proportion between the total number of realized edges (defined as at least one alignment spanning ≥20% of the length of the shortest plasmid in a pair), divided by the total number of potential edges. We calculated the number of potential intra- and interlayers edges, *P*_intra_ and *P*_inter_, respectively, as follows. For intra-layer edges:

$${{{{{{{\mathrm{P}}}}}}}}_{{{{{{{{\mathrm{intra}}}}}}}}} = \mathop {\sum }\limits_{{{{{{{\mathrm{c}}}}}}}}^{{{{{{{\mathrm{C}}}}}}}} \left( {\frac{{{{{{{{{\mathrm{N}}}}}}}}_{{{{{{{\mathrm{c}}}}}}}}\left( {{{{{{{{\mathrm{N}}}}}}}}_{{{{{{{\mathrm{c}}}}}}}} - 1} \right)}}{2}} \right),$$

where *N*_*c*_ is the number of plasmids in cow *c*, and there are *C* cows. For inter-layer edges:

$${{{{{{{\mathrm{P}}}}}}}}_{{{{{{{{\mathrm{inter}}}}}}}}} = \left( {\frac{{{{{{{{{\mathrm{N}}}}}}}}\left( {{{{{{{{\mathrm{N}}}}}}}} - 1} \right)}}{2}} \right) - {{{{{{{\mathrm{P}}}}}}}}_{{{{{{{{\mathrm{intra}}}}}}}}},$$

where *N* is the number of state nodes (plasmid-layer combinations) in the network: $N = {\sum }_c^C N_c$.

### Shuffled networks

We permuted the identity of the cows in which plasmids occur, creating 1000 shuffled networks. The algorithm conserved the distribution of edge weights in the network and the number and identity of links between unique plasmids. However, it did not constrain the number of plasmids in a cow or the number of cows a plasmid could occur in. We calculated the density and the ratio of inter- to intralayer edges of each shuffled network as described above for the observed network. We then compared the intra- and interlayer density and ratio of inter:intra-layer density in the observed network to the distribution of values for these metrics in the shuffled networks. To compare module sharing in observed and shuffled networks we calculated z-scores as

$$\frac{{s_{ij}^{obs} - \overline {s_{ij}^{shuff}} }}{{SD\left( {s_{ij}^{shuff}} \right)}}.$$

### Modularity using Infomap

We obtained network partitioning to modules using the infomap algorithm [54, 80] implemented in the infomapecology R package [56]. In brief, infomap minimizes a function called the map equation using a modified and extended Louvain algorithm to partition the network into modules in a way that minimizes the amount of information needed to describe the movements of a random walker across the network. Modules indicate groups of nodes in the network that are more connected to each other than to other nodes. Infomap is a useful tool for analyzing this type of network because it explicitly accounts for multilayer network structure, and is computationally efficient [56]. Because Infomap is based on flow, it is particularly relevant for this study, which aims to look for signatures of gene flow [56]. To determine whether observed modularity and flow were non-random, we applied Infomap with the same parameters as in the observed network on each of the shuffled networks (described above) and compared the distribution of modularity, module characteristics, flow, and characteristics of the largest modules in the shuffled networks to the observed network.

### Statistical analyses

When comparing measures of an observed network to that obtained from shuffled networks, we calculated p values as the proportion of shuffled networks with values greater or lower than the observed value. Comparing observed to shuffled networks in this way is a well-established and common practice in network ecology [81–83]. We calculated all correlations and their p values using the function cor.test in the stats package of program R. We used Kendall’s tau to measure all correlations due to non-normality of the data and the presence of outliers. We ran Mann-Whitney-Wilcoxon tests using the function wilcox.test in the stats package of program R with “paired” set to “false.”

### Agent-based transmission model

We used Gillespie’s direct method [84] to obtain exact stochastic simulations for our model of gene dispersal. We considered two events. First, the copy of the gene in any state node (a plasmid in a given layer) could be lost at random. Gene loss was determined by a per capita loss rate, and therefore for each loss event, we chose one gene for removal among the state nodes containing the gene, with equal probability. Second, any pair of state nodes could be in contact and spread the gene from one state node to another. For simplicity, we considered a constant contact rate (contacts per time unit) across all state nodes. For each contact event, the algorithm selects two state nodes at random. If one of the state nodes contains the gene, it can transmit to the other state node with a probability equal to the similarity between both state nodes. This explicitly incorporates network structure into the model. Each simulation of the model corresponds to a single realization of this stochastic process. For each of the 20 starting plasmids, we ran the model for 1000 unitless time steps 300 times for each unique combination of contact and loss rate.

### Code

All data management and analysis were conducted in R v.4.1.1 [85].

## Supplementary information

Supplemental material

## Data Availability

All data files and R scripts used for statistical analysis and generating figures for this work are available on the GitHub repository: https://github.com/Ecological-Complexity-Lab/Plasmid_multilayer_networks
