# Supplementary material for: Multilayer networks of plasmid genetic similarity reveal potential pathways of gene transmission
Source: ISME J. 2023 Feb 9;17(5):649–59. doi: 10.1038/s41396-023-01373-5 (PMC10119158; doi:10.1038/s41396-023-01373-5)
Supplement: Supplementary file 1 — Supplemental material [file 41396_2023_1373_MOESM1_ESM.pdf]

Supplementary Information

Super-spreading and degree distribution

The degree distribution of the network led us to ask if it might display scale-free behavior [1], in which the degree of nodes decreases according to the power law, following $P(k) \approx k^{-\gamma}$ where k is the degree and $2 < \gamma < 3$. This topology has been observed in other networks, including those based on genetic similarity between plasmids [2]. A scale-free network is a signature of preferential attachment in which highly connected nodes become more connected and indicates little ecological or evolutionary constraints on the plasmids that are highly connected [1], as has been shown in other ecological networks [2–4]. To investigate whether our network may be scale-free, we first plotted the degree against its probability on a log-log scale (see figure below) and then tested the fit of the power law to the data using the function fit_power_law in the package igraph with implementation "plfit", which calculates the minimum value at which power law behavior is observed in the data [5] and tests the null hypothesis that the data come from a power law distribution using the Kolmogorov-Smirnov statistic [5, 6]. While the network as a whole did not display scale-free behavior ($\gamma = 1.68$), the tail of the distribution (degree ≥ 8) appeared to ($\gamma = 2.75$).

However, a fit to a particular distribution does not mean it is the best fitting distribution and therefore we then compared the fit of this tail of the distribution to the power law distribution with fits to three alternative distributions (discrete log-normal, discrete exponential, discrete Poisson) [6] using the Vuong test implemented with the function compare_distributions in the package poweRlaw in R [7]. This function uses a log-likelihood ratio as the test statistic for the null hypothesis that the two compared distributions provide equally good fits to the data. We use a two-sided p-value that indicates the probability of obtaining a log-likelihood ratio that deviates as far from zero as the observed value if the two compared distributions are equally good. A positive test statistic and p-value < 0.1 indicates that the power law distribution provides a better fit to the data while a negative test statistic and p-value > 0.1 indicates that the power law does not provide a better fit than the alternative distribution. We found that the power law did not provide a better fit than the log-normal distribution [6] (See table below).

References

1. Barabasi AL, Albert R. Emergence of scaling in random networks. *Science* 1999; **286**: 509–512.
2. Fondi M, Fani R. The horizontal flow of the plasmid resistome: clues from inter-generic similarity networks. *Environ Microbiol* 2010; **12**: 3228–3242.
3. Jordano P, Bascompte J, Olesen JM. Invariant properties in coevolutionary networks of plant-animal interactions. *Ecol Lett* 2002; **6**: 69–81.
4. Yamashita A, Sekizuka T, Kuroda M. Characterization of Antimicrobial Resistance Dissemination across Plasmid Communities Classified by Network Analysis. *Pathogens* 2014; **3**: 356–376.
5. Csardi G, Nepusz T. The Igraph Software Package for Complex Network Research. 2005; **Complex Systems**: 1695.
6. Clauset A, Shalizi CR, Newman MEJ. Power-Law Distributions in Empirical Data. *SIAM Rev* 2009; **51**: 661–703.
7. Gillespie CS. Fitting Heavy Tailed Distributions: The poweRlaw Package. *J Stat Softw* 2015; **64**: 1–16.

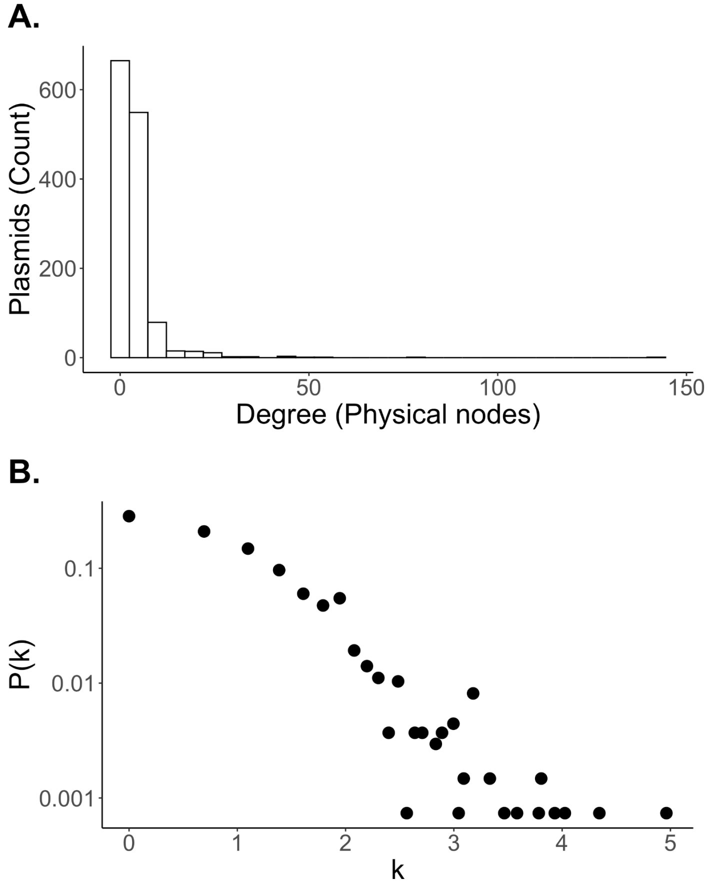

A. Degree distribution of physical nodes. The number of links of unique plasmids (degree of physical nodes) ranged from 1 - 143 with a mean of 4.1 and median of 3. B. Log-log plot of the degree *k* and frequency of each degree P(k). The number of inter-layer links per plasmid ranged from 1 - 13 but 92.5% of plasmids had links to only one other layer.

Comparison of the fit of the power law to three alternative distributions (discrete log-normal, discrete exponential, and discrete Poisson) to the data set. The test statistic is a log-likelihood ratio. We use a two-sided p-value that indicates the probability of obtaining a log-likelihood ratio that deviates as far from zero as the observed value if the two distributions are equally good. A positive test statistic and p-value < 0.1 indicates that the power law distribution provides a better fit to the data while a negative test statistic and p-value > 0.1 indicates that the power law does not provide a better fit than the alternative distribution.

Comparison	Log likelihood ratio for alternative distribution	p-value (two-sided)
Power law - Discrete log-normal	-0.44	0.66
Power law - Discrete exponential	1.66	0.10
Power law - Discrete Poisson	2.23	0.03

Supplementary Figures

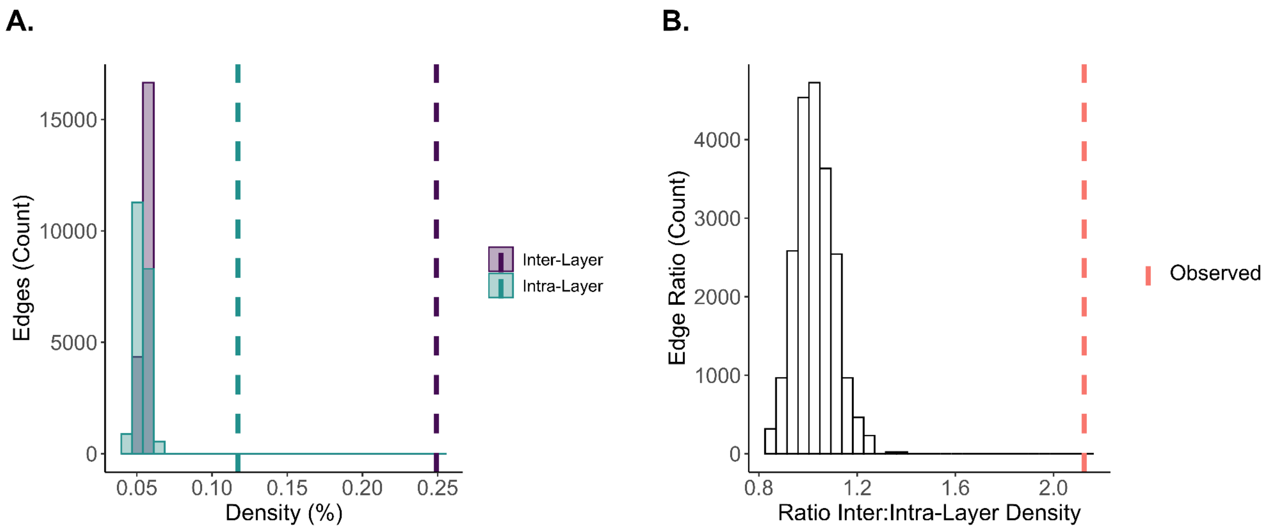

Supplementary Figure 1. Comparison of: (A.) density: the percent of potential inter- and intra-layer edges realized and (B.) the ratio of realized potential inter- to intra-layer edges in the observed (dashed vertical lines) and 1,000 shuffled networks (histograms). Shuffled networks were obtained by permuting layer identities.

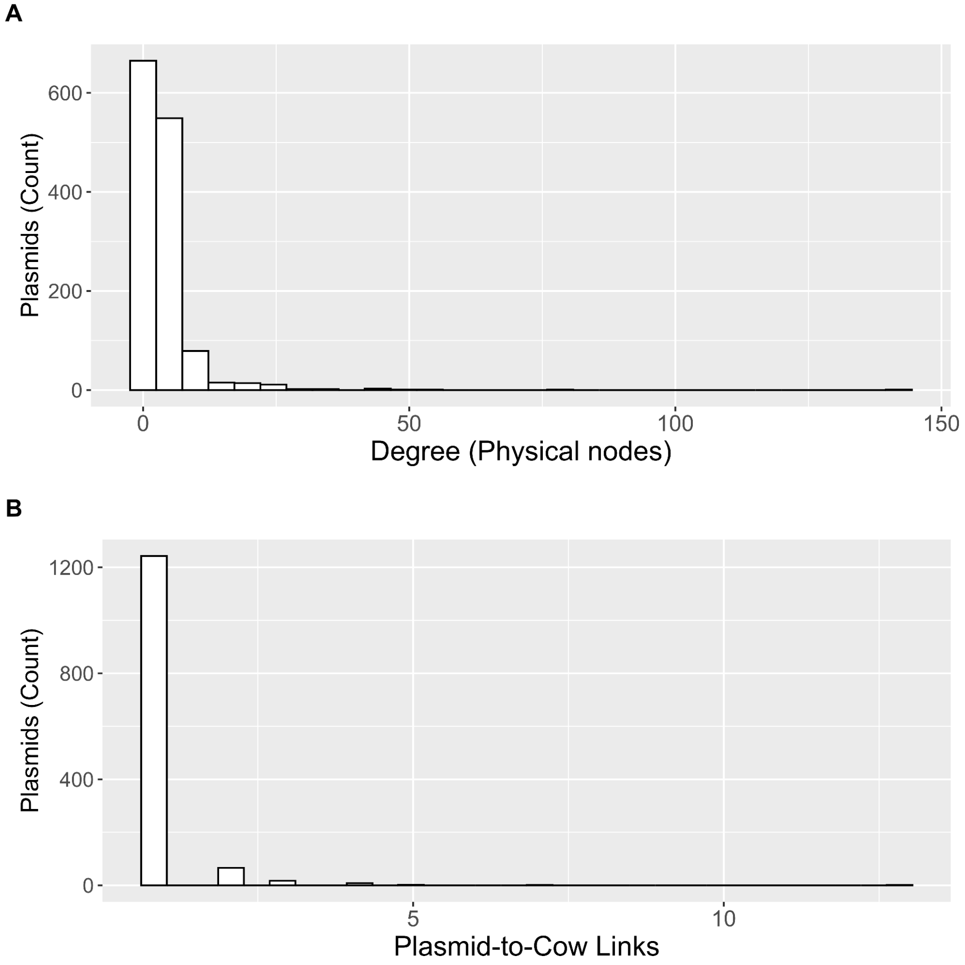

Supplementary Figure 2. Histogram showing the distribution of: (A) degree of plasmids (physical nodes); (B) links to cows for each plasmid.

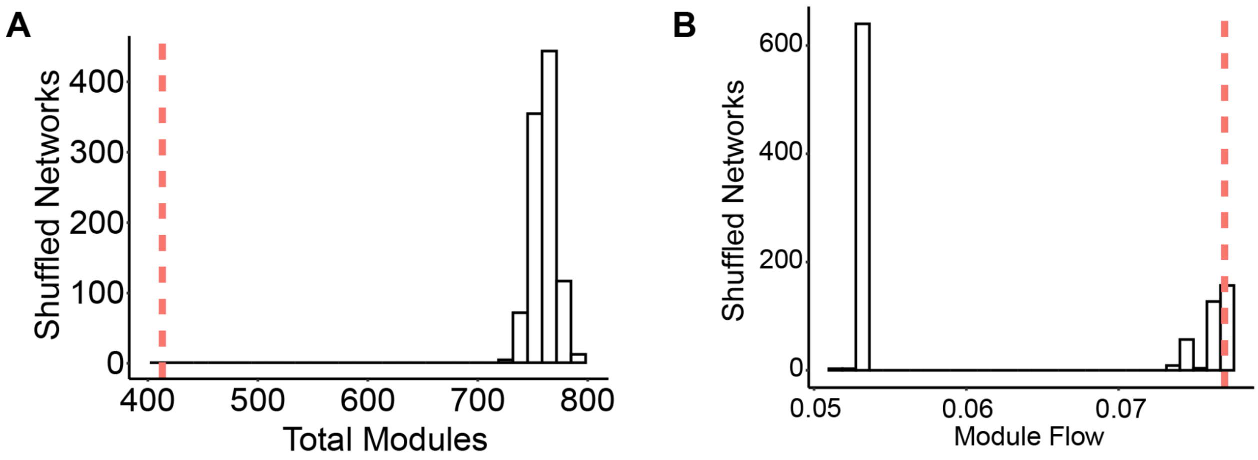

Supplementary Figure 3. (A) Comparison of the total number of modules between the observed and 1 000 shuffled networks. (B) Comparison of flow within the largest module of the observed and shuffled networks. In both panels the vertical dashed line indicates the value of the observed network.

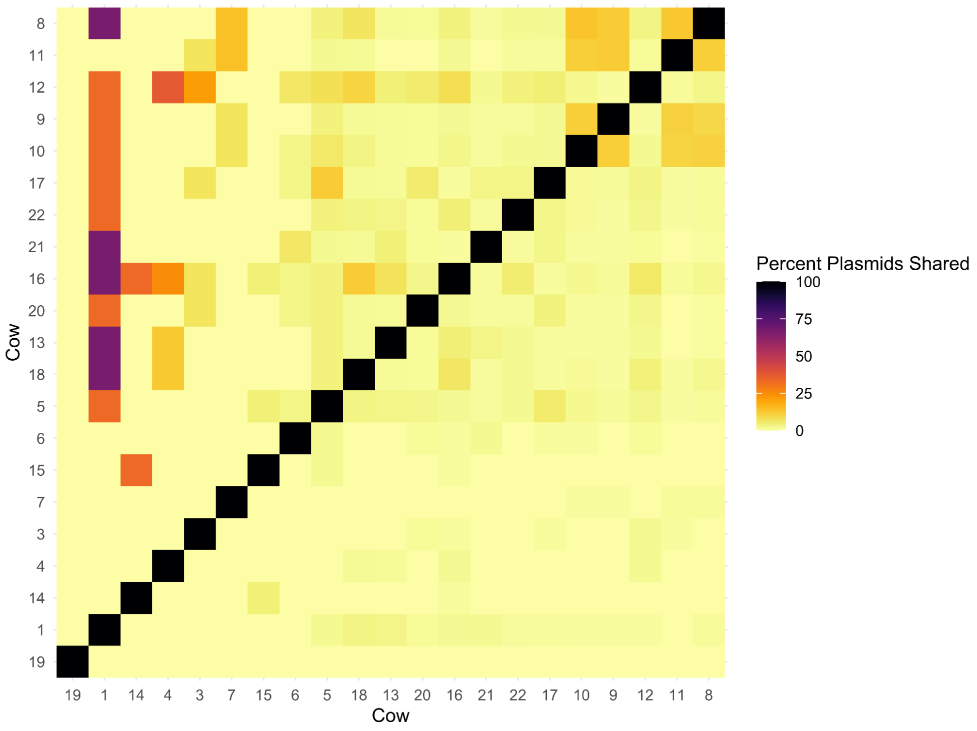

Supplementary Figure 4. Comparison of the percent of plasmids shared between cows *i* and *j*. The number of plasmids per cow ranged from 1 - 175, with a median of 67. The matrices are asymmetric. Each cell is calculated as: the number of plasmids that cow *j* shares with *i*, divided by the total number of plasmids that *i* has.

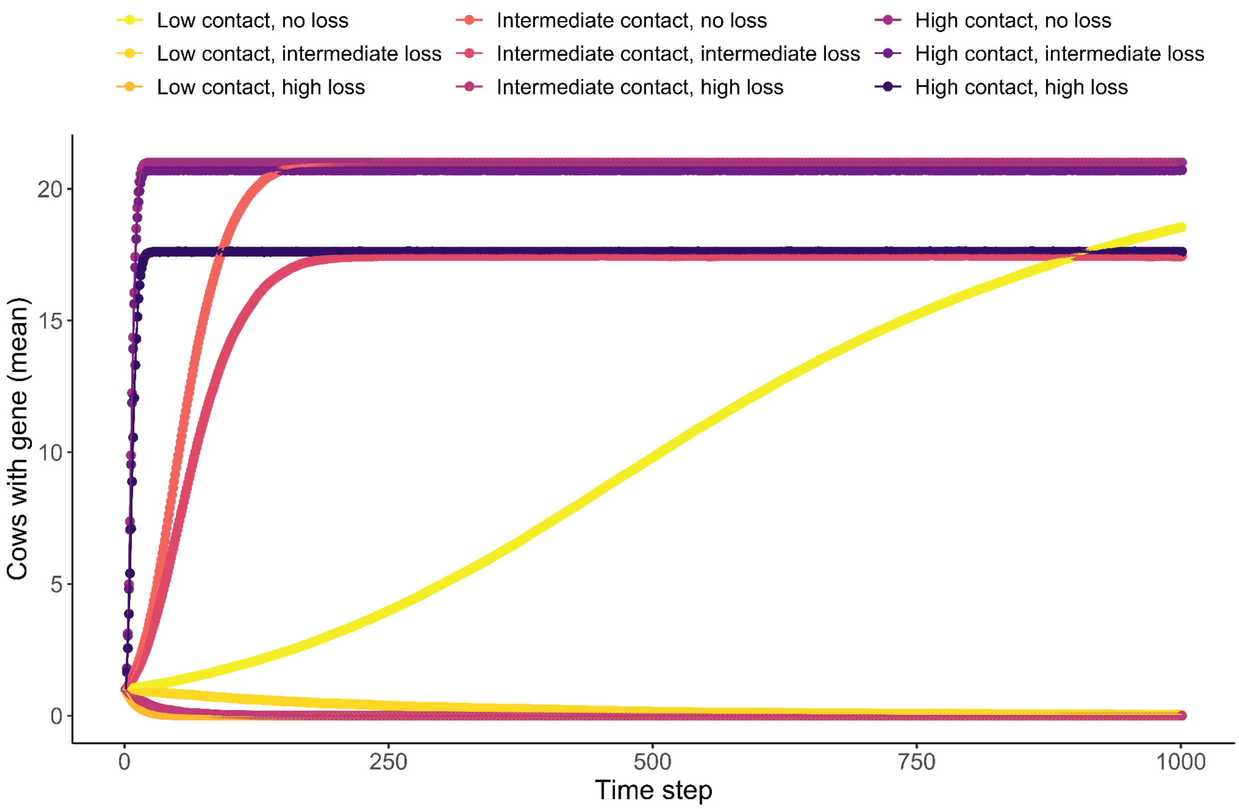

Supplementary Figure 5. Simulated gene transmission dynamics in a cow population. Results of simulations of gene transmission among cows when the gene originates in a peripheral plasmid. Each point is the number of cows with the gene at each time step averaged over 300 simulations per plasmid. Contact refers to the contact rate between plasmids. When plasmids encounter each other, and consequently exchange genes, at high rates, the gene is quickly transmitted to all the cow population.

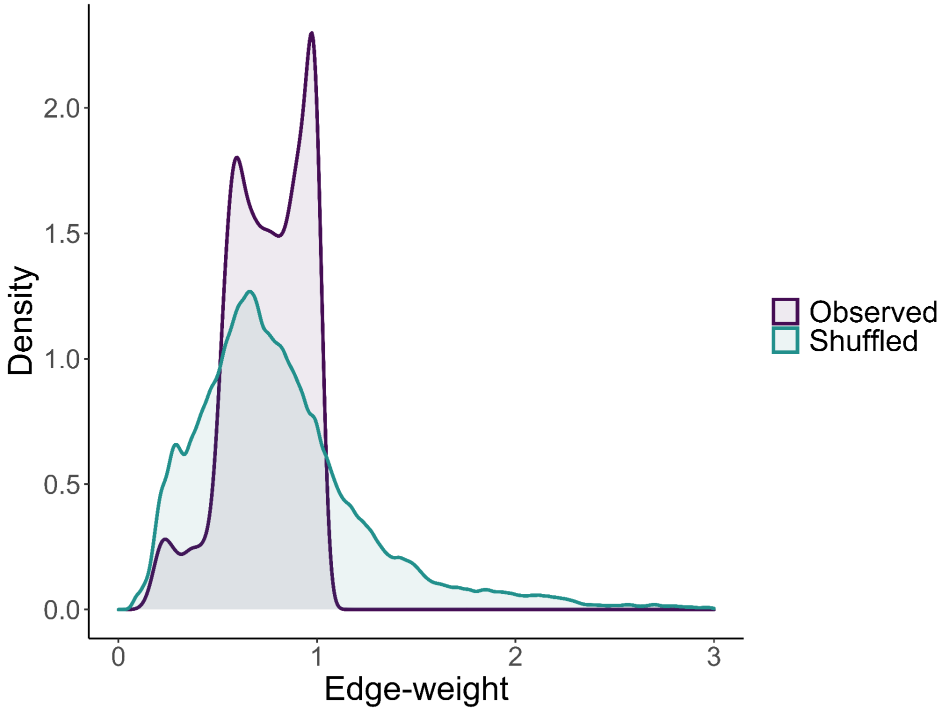

Supplementary Figure 6. Comparison of the distribution of edge-weights between the observed network (purple) and shuffled networks (teal). Note in shuffled networks that the alignment length could be longer than the length of either plasmid in a pair, which is not possible in the observed data.

A.

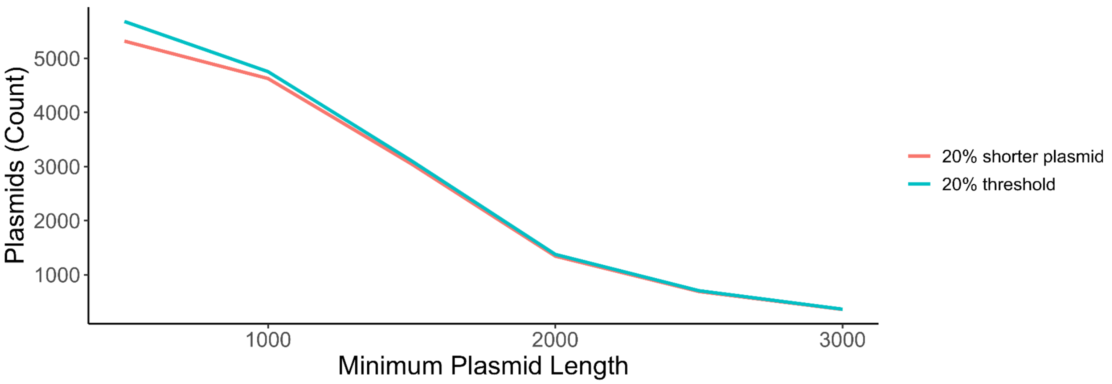

B.

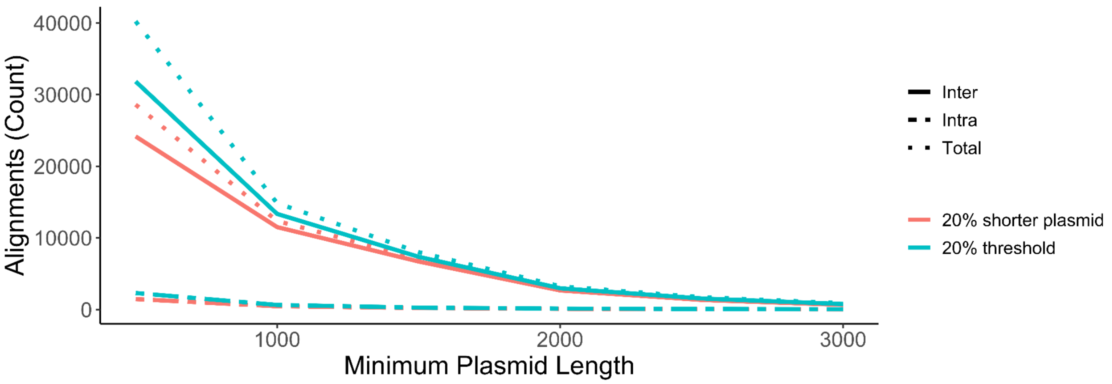

Supplementary Figure 7. Effect of thresholds for plasmid length and alignment length on the number of: (A.) plasmids and (B.) alignments retained in the data set.

Supplementary Tables

Supplementary Table 1. Results of simulation model for central and peripheral cows at each contact and loss rate showing the percent of the 300 simulations in which the gene reached all 21 cows and, in the case that all cows were reached, the mean number of time steps required.

Plasmid	Contact rate	Loss rate	Cows with gene (%)	Time steps
Highly-connected	1000	0	100	14.2
	1000	0.01	98.7	14.4
	1000	0.1	85.5	16.3
	100	0	100	127.9
	100	0.01	85.5	148.8
	100	0.1	0	-
	10	0	14.7	899.5
	10	0.01	0	-
	10	0.1	0	-
Peripheral	1000	0	100	14.2
	1000	0.01	98.6	14.4
	1000	0.1	84.5	16.5
	100	0	100	127.6
	100	0.01	83.7	148.3
	100	0.1	0	-
	10	0	15.6	903.2
	10	0.01	0	-
	10	0.1	0	-

Supplementary Table 3. Results of statistical comparisons of the number of state nodes, physical nodes, and layers per module in each subnetwork (horizontal gene transfer, recent dispersal, distant dispersal). We first compare all subnetworks together with a Kruskall-Wallis test and then perform pair-wise comparisons between each subnetwork with a Dunn test and Bonferroni correct. We specify the comparison, network metric, statistical test used, test value, and p-value. Significant p-values (< 0.05) are highlighted in bold.

Comparison	Network metric (per module)	Statistical test	Statistical measure	Value	p-value
Overall	State nodes	Kruskall-Wallis	Chi-squared	26.6	**0.000002***
	Physical nodes			60.8	**6.37e-14***
	Layers			29.5	**3.86e-07***
	Module flow			359.51	**< 2.2e-16**
Recent dispersal X HGT	State nodes	Dunn test	Z-score	3.42	**0.002***
	Physical nodes			4.19	**0.0001***
	Layers			3.26	**0.003***
	Module flow			5.27	**4.14e-07***
Recent dispersal X Distant dispersal	State nodes			4.94	**0.000002***
	Physical nodes			7.73	**3.22e-14***
	Layers			5.32	**3.11e-07***
	Module flow			-14.26	**1.24e-45***
HGT x Distant dispersal	State nodes			-0.26	1
	Physical nodes			0.84	1
	Layers			0.17	1
	Module flow			-15.5	**1.02e-53***

Conserved domain analysis

Module #1 - the large module

Plasmid 141

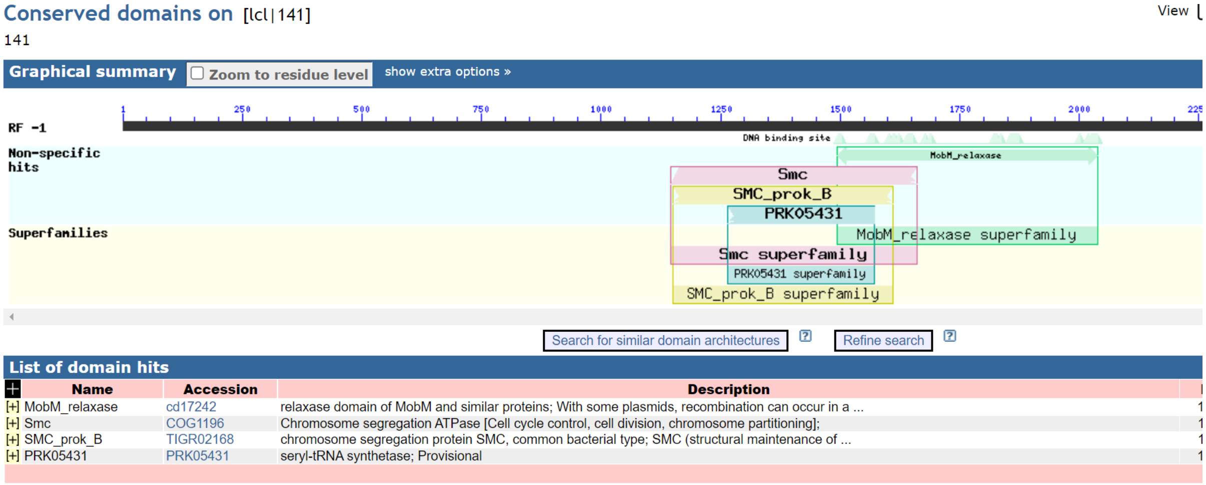

Name	Accession	Description
[+] MobM_relaxase	cd17242	relaxase domain of MobM and similar proteins; With some plasmids, recombination can occur in a ...
[+] Smc	COG1196	Chromosome segregation ATPase [Cell cycle control, cell division, chromosome partitioning];
[+] SMC_prok_B	TIGR02168	chromosome segregation protein SMC, common bacterial type; SMC (structural maintenance of ...
[+] PRK05431	PRK05431	seryl-tRNA synthetase; Provisional

Plasmid 199

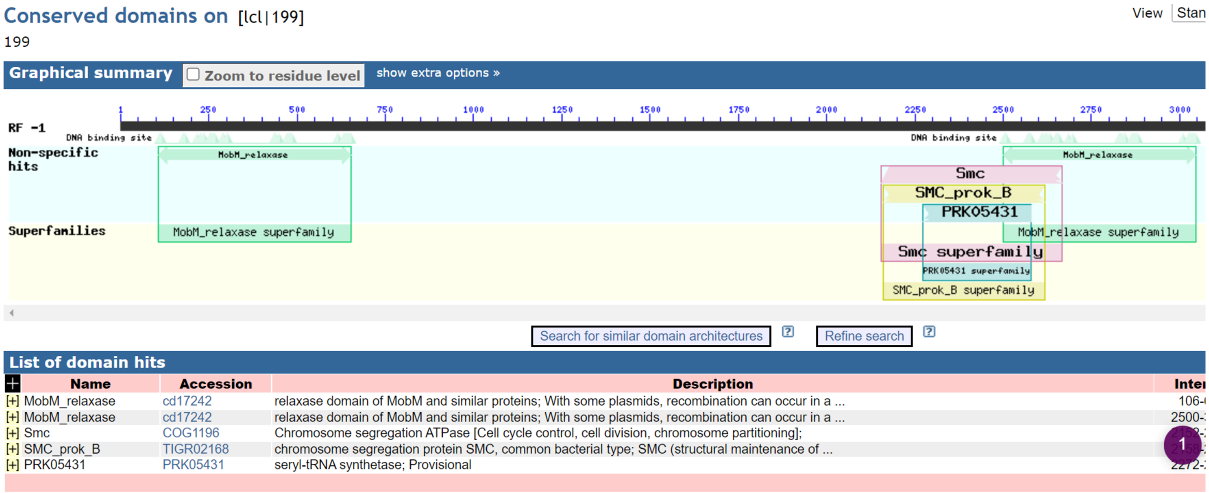

Name	Accession	Description	Inte[illegible]
[+] MobM_relaxase	cd17242	relaxase domain of MobM and similar proteins; With some plasmids, recombination can occur in a ...	106-[illegible]
[+] MobM_relaxase	cd17242	relaxase domain of MobM and similar proteins; With some plasmids, recombination can occur in a ...	2500-[illegible]
[+] Smc	COG1196	Chromosome segregation ATPase [Cell cycle control, cell division, chromosome partitioning];	[illegible]
[+] SMC_prok_B	TIGR02168	chromosome segregation protein SMC, common bacterial type; SMC (structural maintenance of ...	[illegible]
[+] PRK05431	PRK05431	seryl-tRNA synthetase; Provisional	2272-[illegible]

Plasmid 294

Conserved domains on [lcl|294]

View

294

Graphical summary ☐ Zoom to residue level   show extra options »

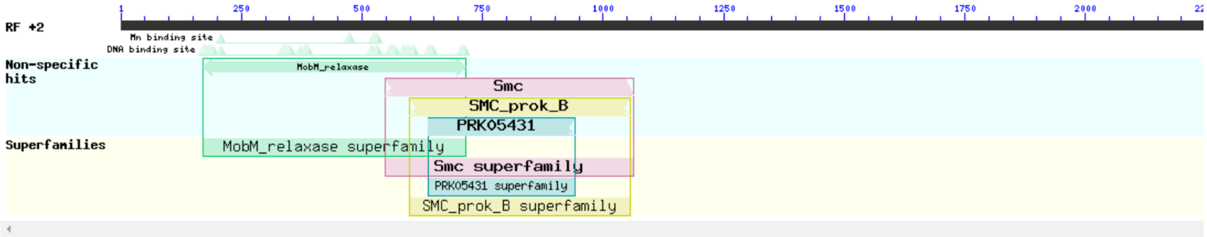

Search for similar domain architectures   Refine search

List of domain hits

	Name	Accession	Description
[+]	MobM_relaxase	cd17242	relaxase domain of MobM and similar proteins; With some plasmids, recombination can occur in a ...
[+]	Smc	COG1196	Chromosome segregation ATPase [Cell cycle control, cell division, chromosome partitioning];
[+]	SMC_prok_B	TIGR02168	chromosome segregation protein SMC, common bacterial type; SMC (structural maintenance of ...
[+]	PRK05431	PRK05431	seryl-tRNA synthetase; Provisional

Plasmid 415

Conserved domains on [lcl|415]

View S

415

Graphical summary ☐ Zoom to residue level   show extra options »

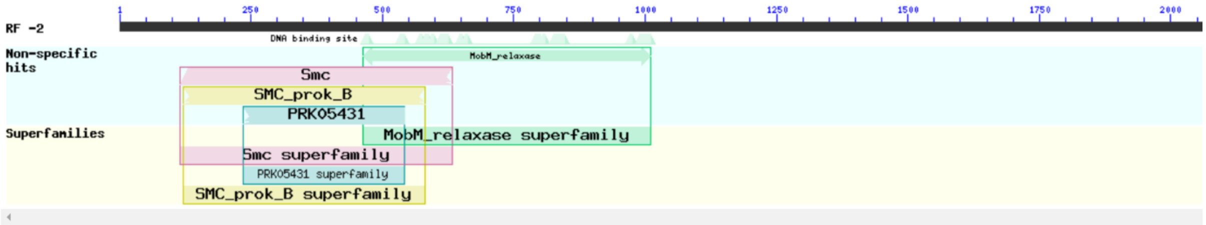

Search for similar domain architectures   Refine search

List of domain hits

	Name	Accession	Description	I
[+]	MobM_relaxase	cd17242	relaxase domain of MobM and similar proteins; With some plasmids, recombination can occur in a ...	4
[+]	Smc	COG1196	Chromosome segregation ATPase [Cell cycle control, cell division, chromosome partitioning];	
[+]	SMC_prok_B	TIGR02168	chromosome segregation protein SMC, common bacterial type; SMC (structural maintenance of ...	[illegible]
[+]	PRK05431	PRK05431	seryl-tRNA synthetase; Provisional	

Plasmid 1486

Conserved domains on [lcl|1486]

View Standard

1486

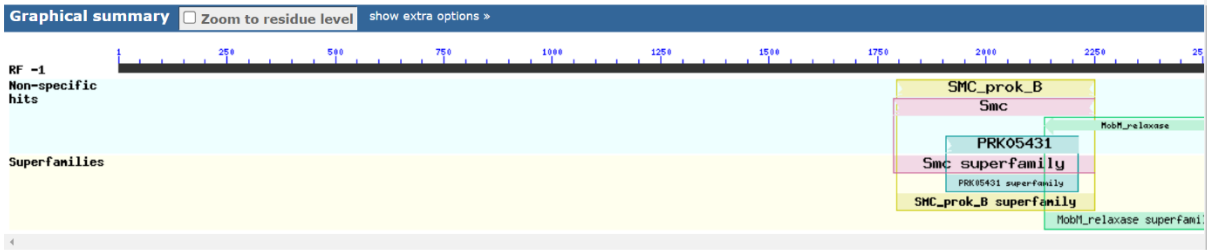

Search for similar domain architectures | Refine search

List of domain hits

Name	Accession	Description	Interval
[+] SMC_prok_B	TIGR02168	chromosome segregation protein SMC, common bacterial type; SMC (structural maintenance of ...	1792-2250
[+] Smc	COG1196	Chromosome segregation ATPase [Cell cycle control, cell division, chromosome partitioning];	1786-2250
[+] MobM_relaxase	cd17242	relaxase domain of MobM and similar proteins; With some plasmids, recombination can occur in a ...	2134-2559
[+] PRK05431	PRK05431	seryl-tRNA synthetase; Provisional	1906-2211

Plasmid 3582

3582

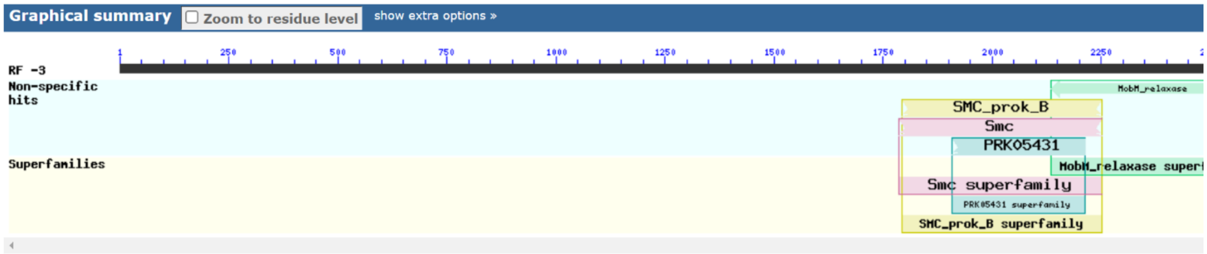

Search for similar domain architectures | Refine search

List of domain hits

Name	Accession	Description	Inter
[+] MobM_relaxase	cd17242	relaxase domain of MobM and similar proteins; With some plasmids, recombination can occur in a ...	2134-2
[+] SMC_prok_B	TIGR02168	chromosome segregation protein SMC, common bacterial type; SMC (structural maintenance of ...	1792-2
[+] Smc	COG1196	Chromosome segregation ATPase [Cell cycle control, cell division, chromosome partitioning];	1786-2
[+] PRK05431	PRK05431	seryl-tRNA synthetase; Provisional	1906-2

Plasmid 4619

4619

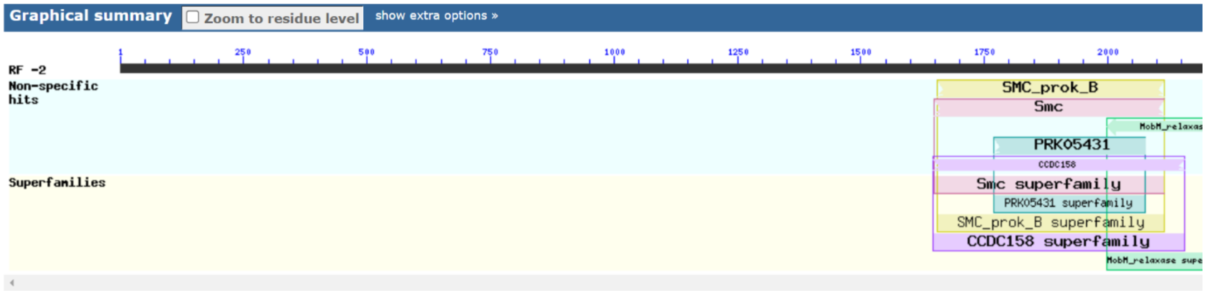

Search for similar domain architectures | Refine search

List of domain hits

Name	Accession	Description	Inter
[+] SMC_prok_B	TIGR02168	chromosome segregation protein SMC, common bacterial type; SMC (structural maintenance of ...	1655-[illegible]
[+] Smc	COG1196	Chromosome segregation ATPase [Cell cycle control, cell division, chromosome partitioning];	1649-[illegible]
[+] MobM_relaxase	cd17242	relaxase domain of MobM and similar proteins; With some plasmids, recombination can occur in a ...	1997-[illegible]
[+] PRK05431	PRK05431	seryl-tRNA synthetase; Provisional	1769-[illegible]
[+] CCDC158	pfam15921	Coiled-coil domain-containing protein 158; CCDC158 is a family of proteins found in eukaryotes. ...	1646-[illegible]

Plasmid 5335

Conserved domains on [lcl|5335]

View

5335

Graphical summary ☐ Zoom to residue level show extra options »

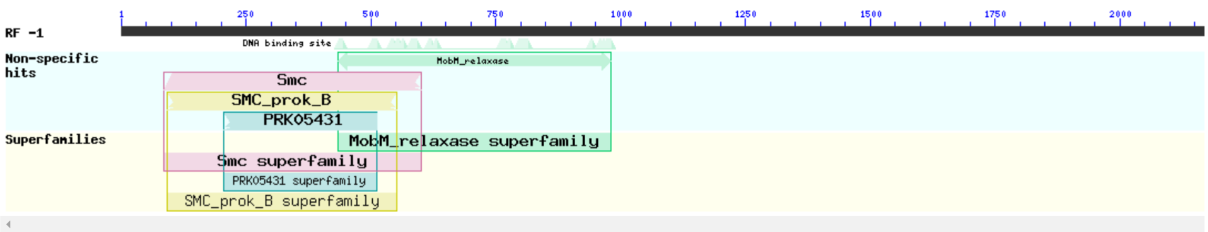

Search for similar domain architectures | Refine search

List of domain hits

Name	Accession	Description
[+] MobM_relaxase	cd17242	relaxase domain of MobM and similar proteins; With some plasmids, recombination can occur in a ...
[+] Smc	COG1196	Chromosome segregation ATPase [Cell cycle control, cell division, chromosome partitioning];
[+] SMC_prok_B	TIGR02168	chromosome segregation protein SMC, common bacterial type; SMC (structural maintenance of ...
[+] PRK05431	PRK05431	seryl-tRNA synthetase; Provisional

Plasmid 6072

Conserved domains on [lcl|6072]

View

6072

Graphical summary ☐ Zoom to residue level show extra options »

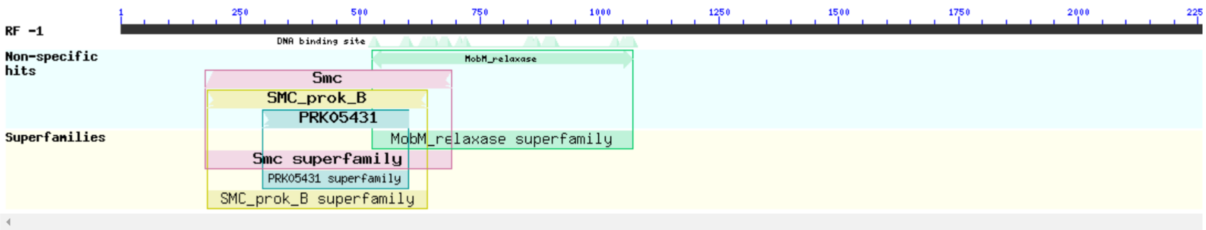

Search for similar domain architectures | Refine search

List of domain hits

Name	Accession	Description
[+] MobM_relaxase	cd17242	relaxase domain of MobM and similar proteins; With some plasmids, recombination can occur in a ...
[+] Smc	COG1196	Chromosome segregation ATPase [Cell cycle control, cell division, chromosome partitioning];
[+] SMC_prok_B	TIGR02168	chromosome segregation protein SMC, common bacterial type; SMC (structural maintenance of ...
[+] PRK05431	PRK05431	seryl-tRNA synthetase; Provisional

Plasmid 6075

Conserved domains on [lcl|6075]

View | S

6075

Graphical summary ☐ Zoom to residue level show extra options »

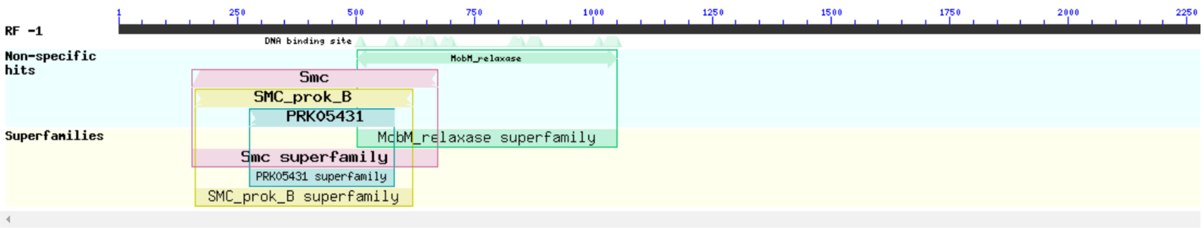

Search for similar domain architectures | Refine search

List of domain hits

Name	Accession	Description	I
[+] MobM_relaxase	cd17242	relaxase domain of MobM and similar proteins; With some plasmids, recombination can occur in a ...	5
[+] Smc	COG1196	Chromosome segregation ATPase [Cell cycle control, cell division, chromosome partitioning];	1
[+] SMC_prok_B	TIGR02168	chromosome segregation protein SMC, common bacterial type; SMC (structural maintenance of ...	1
[+] PRK05431	PRK05431	seryl-tRNA synthetase; Provisional	2

Plasmid 7250

Conserved domains on [lcl|7250] View Sta

7250

Graphical summary ☐ Zoom to residue level show extra options »

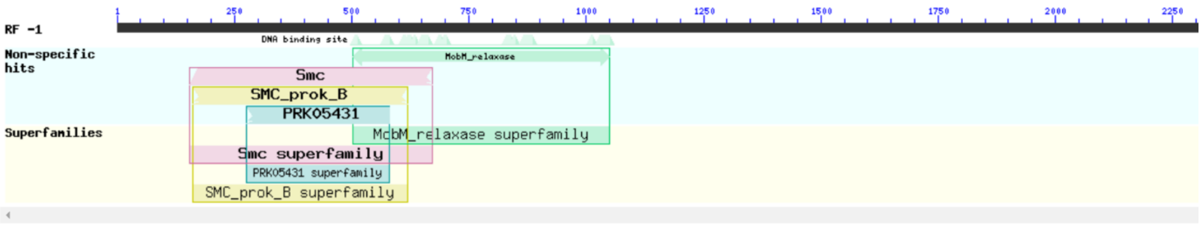

Search for similar domain architectures | Refine search

List of domain hits

Name	Accession	Description	Int
[+] MobM_relaxase	cd17242	relaxase domain of MobM and similar proteins; With some plasmids, recombination can occur in a ...	503
[+] Smc	COG1196	Chromosome segregation ATPase [Cell cycle control, cell division, chromosome partitioning];	155
[+] SMC_prok_B	TIGR02168	chromosome segregation protein SMC, common bacterial type; SMC (structural maintenance of ...	161
[+] PRK05431	PRK05431	seryl-tRNA synthetase; Provisional	275

Plasmid 8165

8165

Graphical summary ☐ Zoom to residue level show extra options »

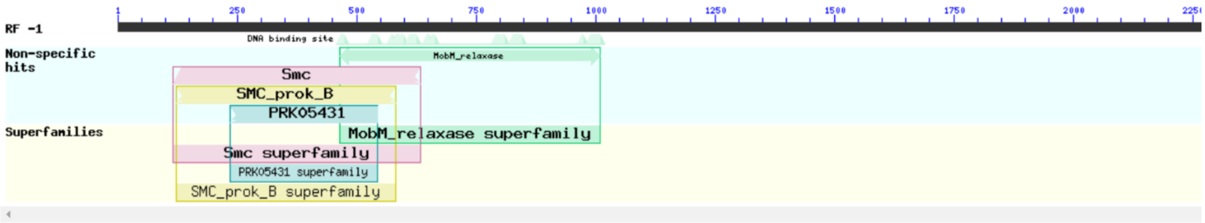

Search for similar domain architectures | Refine search

List of domain hits

Name	Accession	Description	Int
[+] MobM_relaxase	cd17242	relaxase domain of MobM and similar proteins; With some plasmids, recombination can occur in a ...	465
[+] Smc	COG1196	Chromosome segregation ATPase [Cell cycle control, cell division, chromosome partitioning];	117
[+] SMC_prok_B	TIGR02168	chromosome segregation protein SMC, common bacterial type; SMC (structural maintenance of ...	123
[+] PRK05431	PRK05431	seryl-tRNA synthetase; Provisional	237

Module #2 - beta-lactamase

Plasmid 289:

289

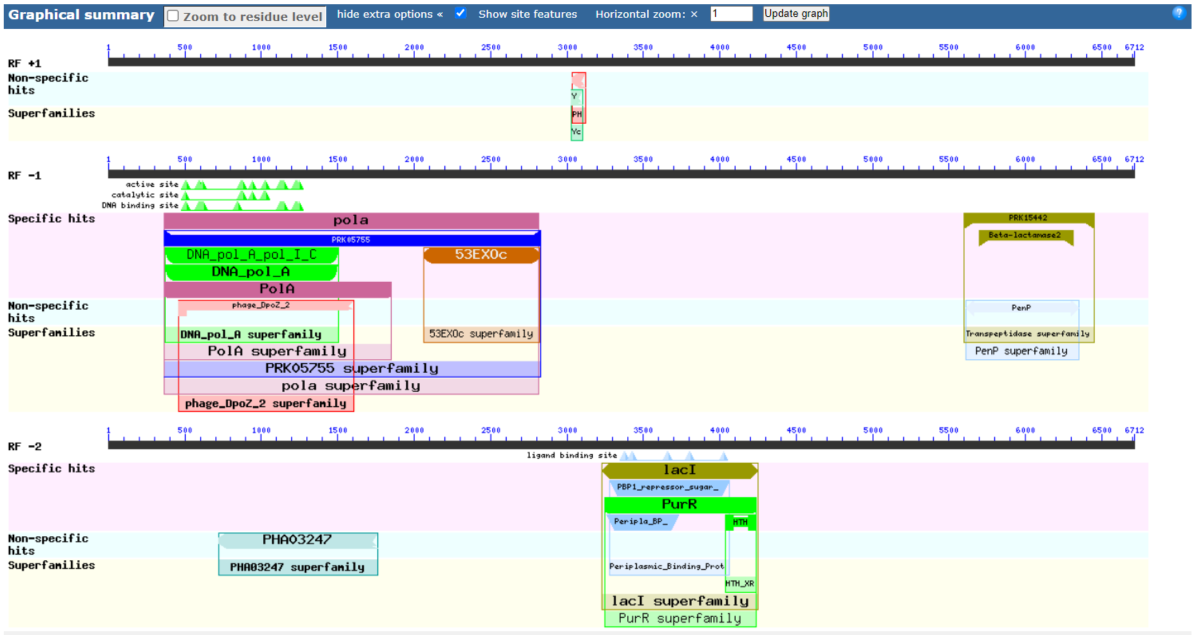

List of domain hits

Name	Accession	Description	Inter
[+] PHP_HisPPase_AMP	cd07438	Polymerase and Histidinol Phosphatase domain of Histidinol phosphate phosphatase (HisPPase) ...	3034-
[+] YciV	COG0613	Predicted metal-dependent phosphoesterase TrpH, contains PHP domain [General function ...	3025-
[+] pola	TIGR00593	DNA polymerase I; All proteins in this family for which functions are known are DNA ...	362-2
[+] PRK05755	PRK05755	DNA polymerase I; Provisional	362-2
[+] DNA_pol_A_pol_I_C	cd08637	Polymerase I functions primarily to fill DNA gaps that arise during DNA repair, recombination ...	371-1
[+] DNA_pol_A	pfam00476	DNA polymerase family A;	368-1
[+] PolA	COG0749	DNA polymerase I - 3'-5' exonuclease and polymerase domains [Replication, recombination and ...	362-1
[+] PRK15442	PRK15442	beta-lactamase TEM; Provisional	5597-
[+] 53EXOc	smart00475	5'-3' exonuclease;	2063-
[+] PenP	COG2367	Beta-lactamase class A [Defense mechanisms];	5609-
[+] Beta-lactamase2	pfam13354	Beta-lactamase enzyme family; This family is closely related to Beta-lactamase, pfam00144, the ...	5696-
[+] phage_DpoZ_2	NF038381	aminoadenine-incorporating DNA polymerase DpoZ;	458-1
[+] lacI	PRK09526	lac repressor; Reviewed	3226-
[+] PBP1_repressor_sugar_binding-like	cd01537	Ligand-binding domain of the LacI-GalR family of transcription regulators and the ...	3277-
[+] PurR	COG1609	DNA-binding transcriptional regulator, LacI/PurR family [Transcription];	3247-
[+] Peripla_BP_3	pfam13377	Periplasmic binding protein-like domain; Thi domain is found in a variety of transcriptional ...	3259-
[+] HTH_LACI	smart00354	helix_turn _helix lactose operon repressor;	4033-
[+] PHA03247	PHA03247	large tegument protein UL36; Provisional	718-1

Plasmid 3952

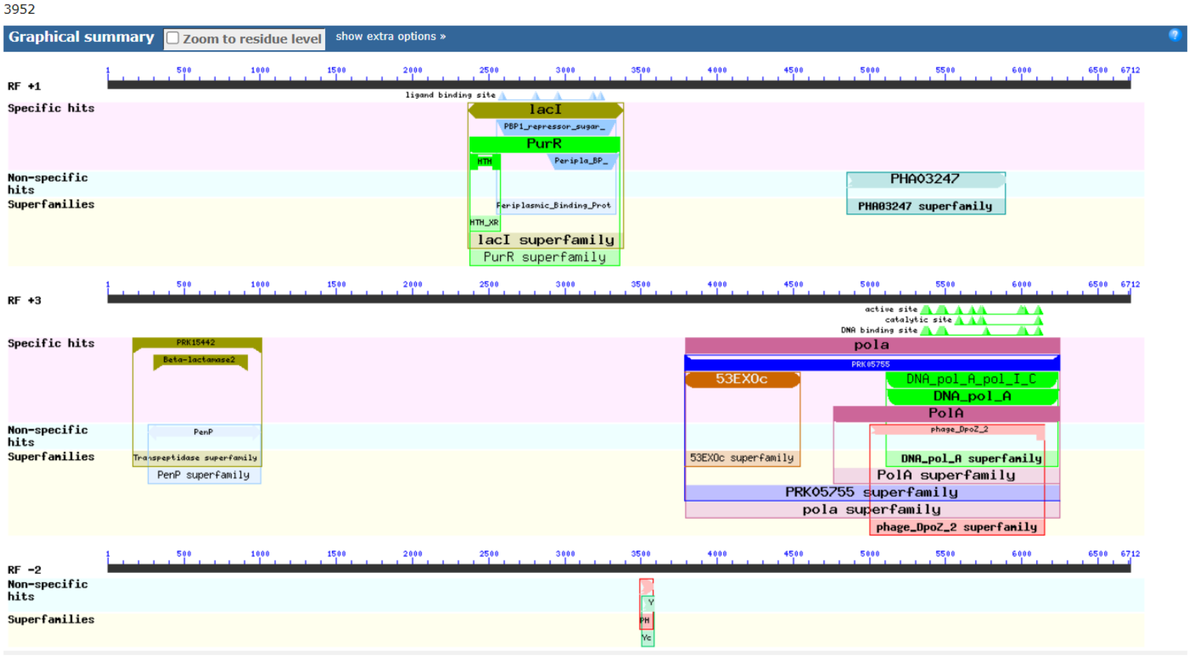

Plasmid 4543

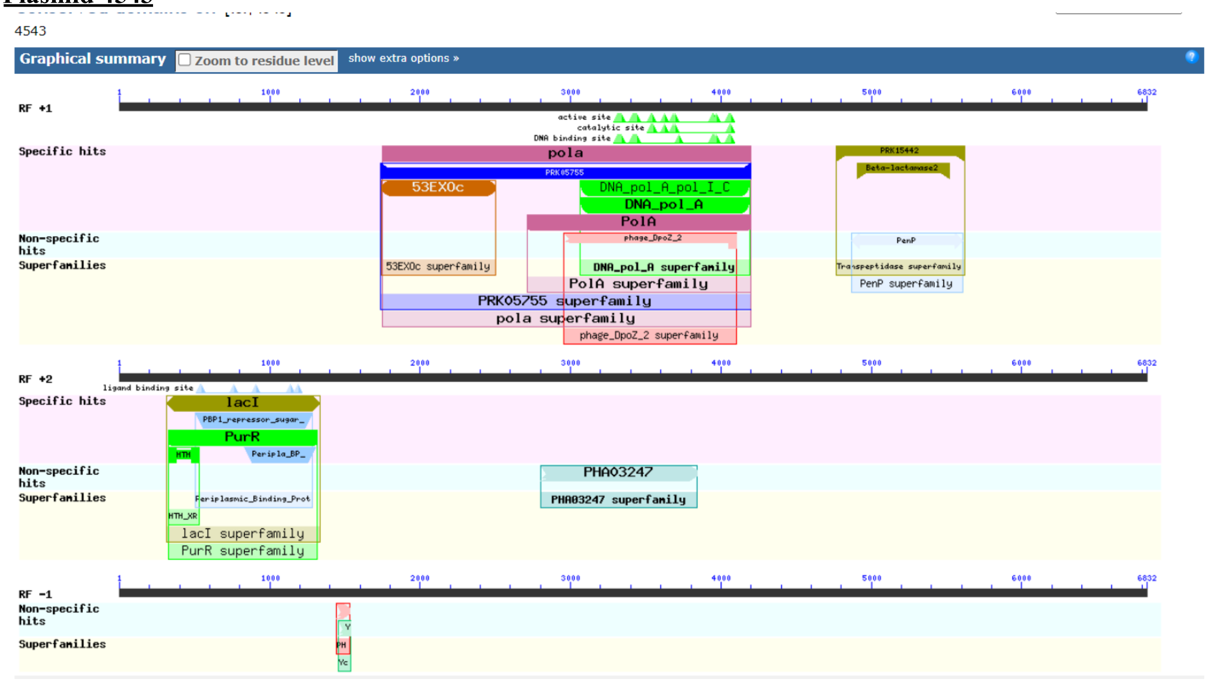

Plasmid 108

108

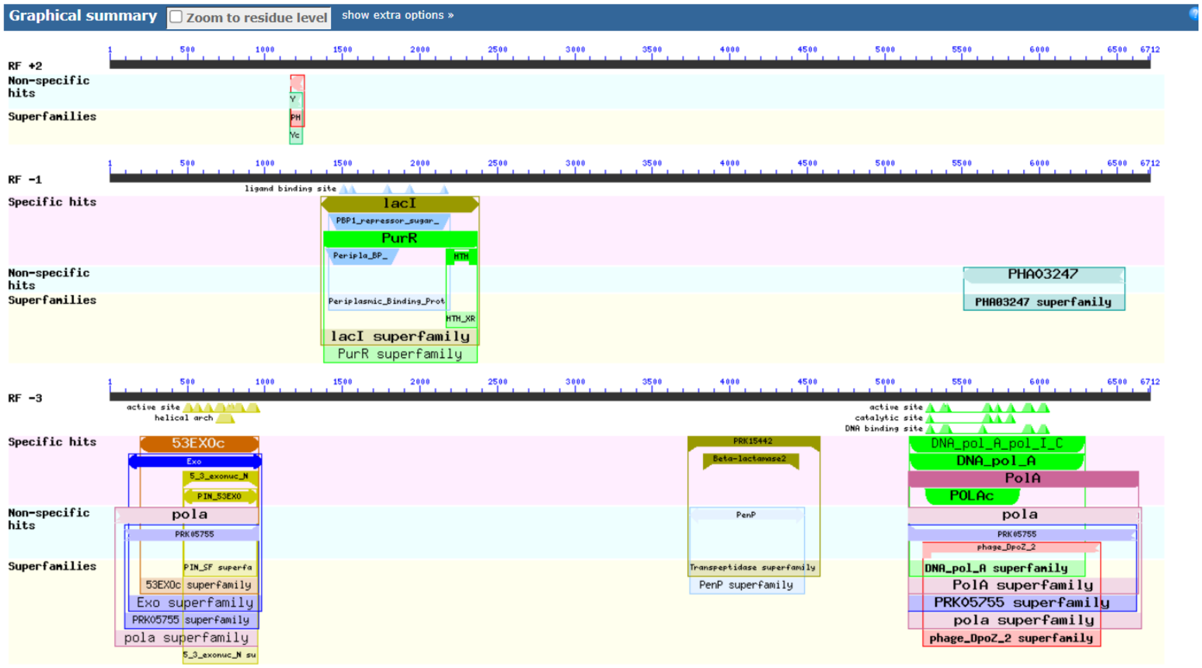

Plasmid 5983

5983

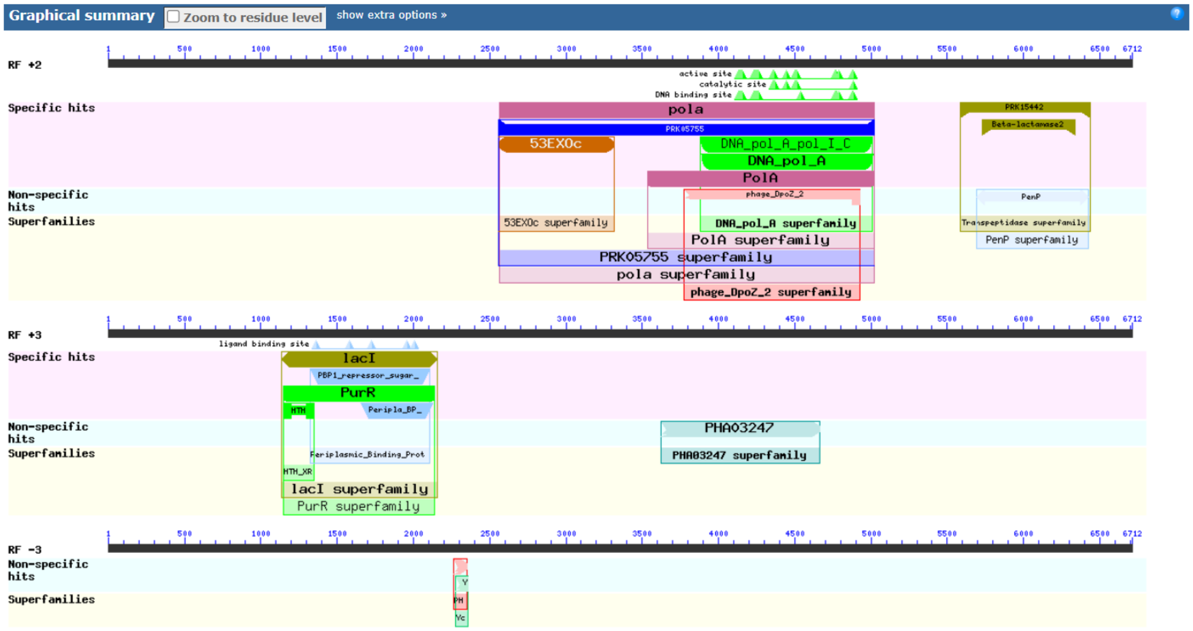

Plasmid 6702

6702

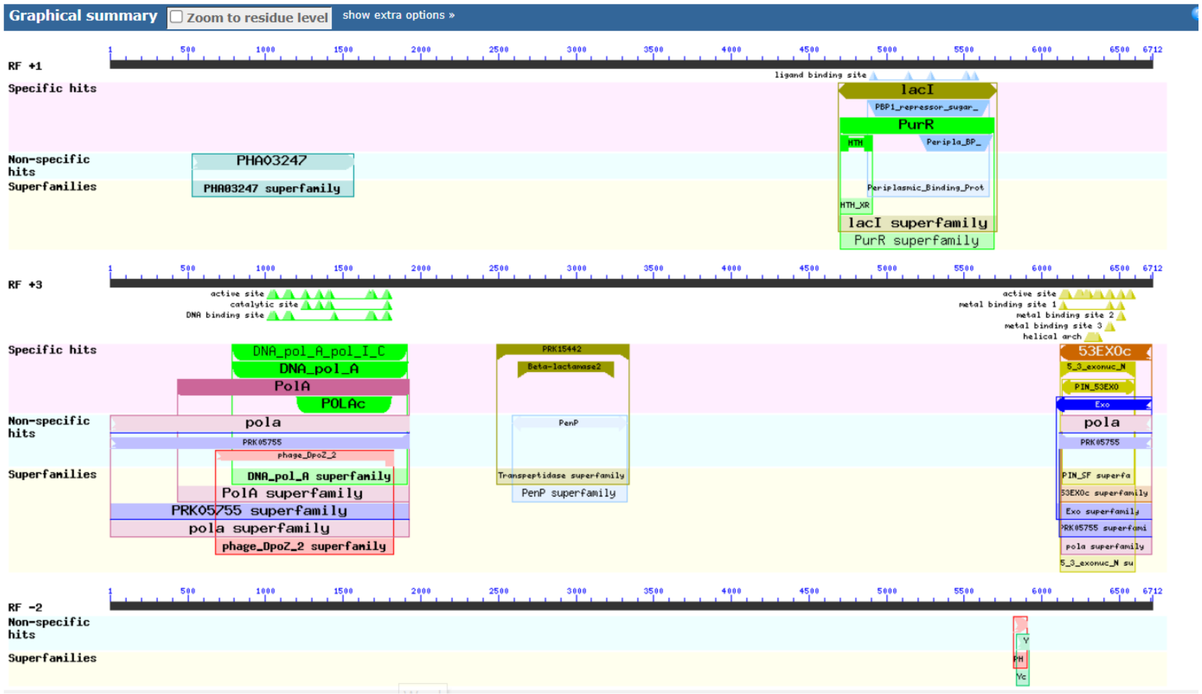

Plasmid 7146

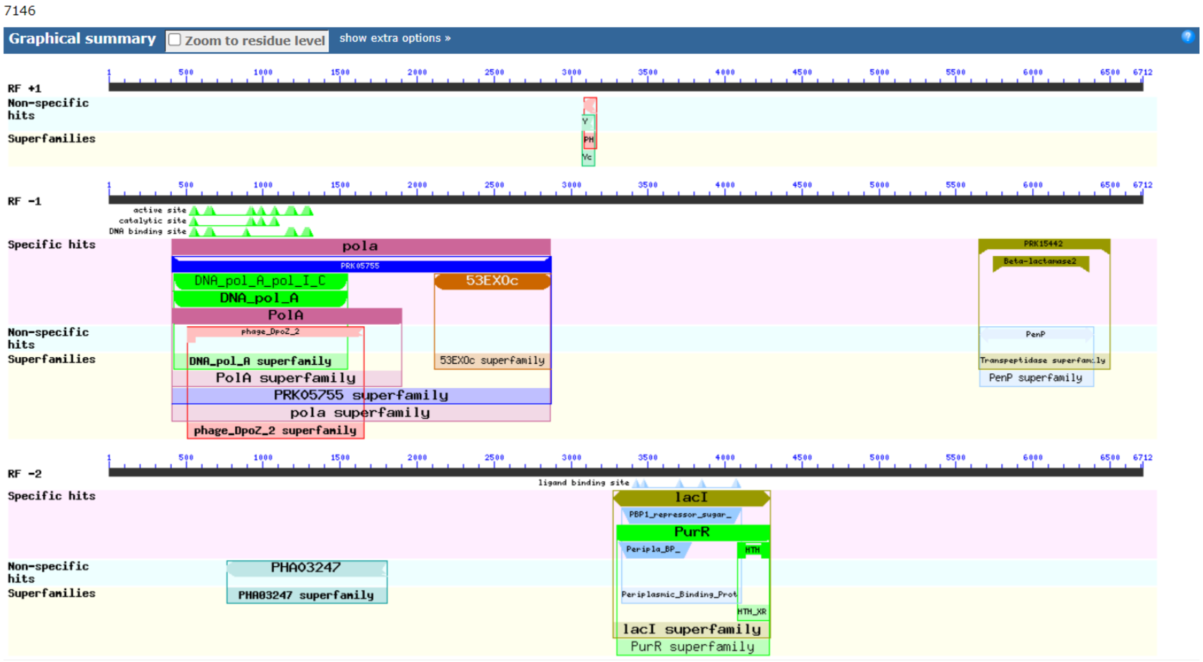

Plasmid 7573

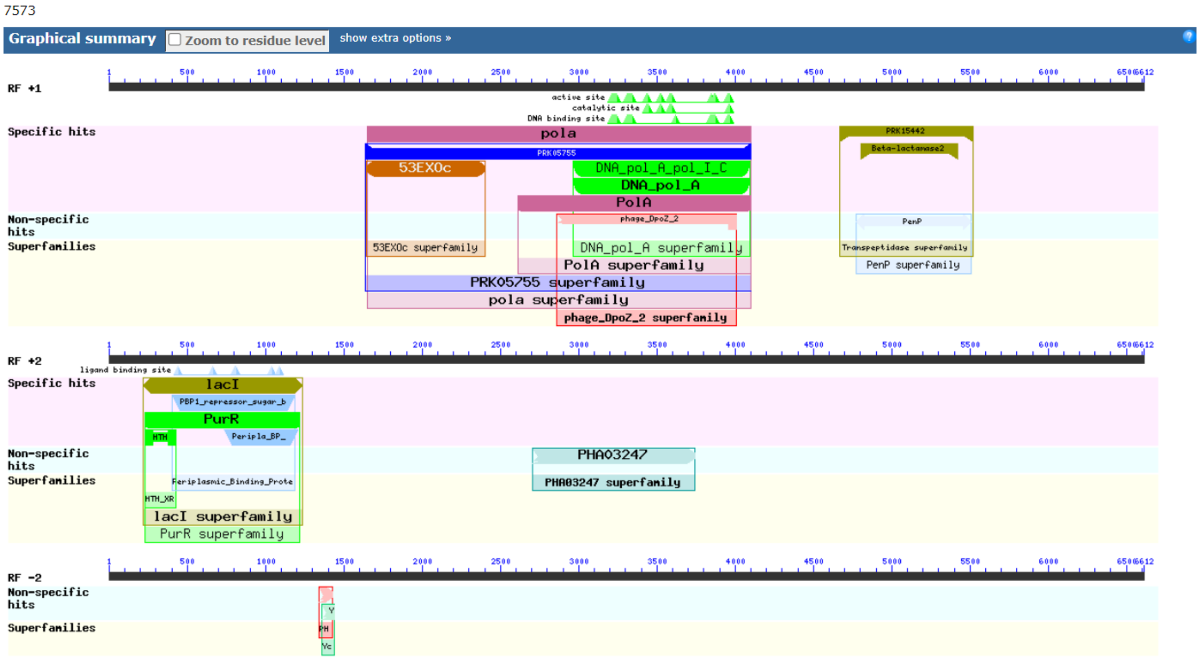

KEGG Orthologies

Supplementary Table 4. An overview of the KEGG orthologies identified. See Supplementary file KOs_in_plasmids.txt on GitHub for a full list of KOs in plasmdis.

KO	annotation
ko:K02335	DNA polymerase I [EC:2.7.7.7]
ko:K02529	LacI family transcriptional regulator
ko:K17836	beta-lactamase class A [EC:3.5.2.6]
ko:K18698	beta-lactamase class A TEM [EC:3.5.2.6]
ko:K03466	DNA segregation ATPase FtsK/SpoIIIE, S-DNA-T family
ko:K19157	mRNA interferase YafQ [EC:3.1.-.-]
ko:K07723	CopG family transcriptional regulator / antitoxin EndoAI
ko:K09803	uncharacterized protein
ko:K07473	DNA-damage-inducible protein J
ko:K18220	ribosomal protection tetracycline resistance protein
ko:K06218	mRNA interferase RelE/StbE

ko:K03496	chromosome partitioning protein
ko:K07474	phage terminase small subunit
ko:K07467	phage replication initiation protein
ko:K06919	putative DNA primase/helicase

Blast results for modules with a high degree (>=20)

There were five plasmids with a hit. Four of these (#289, #108, #3952, #6702) gave the following result:

Plasmids #289, #108, #3952, #6702:

Bacterial expression vector pTrcmelA, complete sequence	Bacterial expre…	7094	7748	62%	0.0	99.87%	5989	LT726808.1
Expression vector pKIL-HIS3, complete sequence	Expression vect…	6907	7692	62%	0.0	99.92%	4806	AF050464.1
Expression vector pTRC-LIC, complete sequence	Expression vect…	6796	7700	62%	0.0	99.89%	6148	EF460847.1
Synthetic construct GtfC gene, complete cds	synthetic constr…	6606	7692	62%	0.0	99.92%	5791	MK802896.1
Cloning vector p6xHis-GFP, complete sequence	unidentified clo…	6606	7692	62%	0.0	99.92%	5271	U89936.1
Cloning vector pTrcHisA-xylB-(teF)xylC-pHluorin, complete sequence	Cloning vector …	6601	7687	62%	0.0	99.89%	6809	MK258733.1
Cloning vector pTrcHisA-xylB-(teF)xylC, complete sequence	Cloning vector …	6601	7687	62%	0.0	99.89%	6075	MK258732.1
Cloning vector pTrcHisA-GFP(teLAA)-(teF)MCP, complete sequence	Cloning vector …	6601	7687	62%	0.0	99.89%	5958	MK258730.1
Cloning vector pTrcHisA-GFP(tvLAA)-(teF)tvmv, complete sequence	Cloning vector …	6601	7687	62%	0.0	99.89%	5826	MK258729.1

First result:
https://www.ncbi.nlm.nih.gov/nucleotide/LT726808.1?report=genbank&log$=nucltop&blast_rank=1&RID=PA4AD9N4013
5' UTR of lacZ, incl. RBS
unnamed protein product; lacI
lacIq promoter, incl. RBS

Second result:
https://www.ncbi.nlm.nih.gov/nucleotide/AF050464.1?report=genbank&log$=nucltop&blast_rank=2&RID=PA4AD9N4013
ampicillin resistance gene beta-lactamase
repressor of lac promoter

Plasmid #4543 gave different blast results:

select all *100 sequences selected* GenBank Graphics Distance tree of results MSA Viewer

Description	Scientific Name	Max Score	Total Score	Query Cover	E value	Per. Ident	Acc. Len	Accession
Cloning vector pKLJ10 DNA, complete sequence	Cloning vector …	4841	5202	41%	0.0	100.00%	4668	LC143902.1
Escherichia sp. Sflu5 cryptic plasmid pAK51	Escherichia sp. …	4841	5202	41%	0.0	100.00%	6511	AM743197.2
Cloning vector pKK388-1, complete sequence	Cloning vector …	4835	5624	44%	0.0	99.96%	5075	U02444.1
Cloning vector pTrc-zwf, complete sequence	Cloning vector …	4828	7973	63%	0.0	99.81%	5629	MH488913.1
Expression vector pKK233-3 containing origin of replication from phage M13	synthetic constr…	4828	5643	44%	0.0	99.92%	4991	X95387.1
Cloning vector pTrc-serB, complete sequence	Cloning vector …	4826	7969	63%	0.0	99.77%	5122	MH488919.1
Cloning vector pTrc-fumB, complete sequence	Cloning vector …	4826	7969	63%	0.0	99.81%	5800	MH488916.1
Cloning vector pTrc-mdh, complete sequence	Cloning vector …	4826	7971	63%	0.0	99.81%	5092	MH488914.1

First hit:
https://www.ncbi.nlm.nih.gov/nucleotide/LC143902.1?report=genbank&log$=nucltop&blast_rank=1&RID=PAN2A2M3013
Cloning vector pKLJ10
L-arabinose regulatory protein
promoter of the L-arabinose operon of E. coli; the araC regulatory gene is transcribed in the opposite direction
beta-lactamase
Rop gene - maintains plasmids at low copy number
